# Phosphoprotein SAK1 is a regulator of acclimation to singlet oxygen in *Chlamydomonas reinhardtii*

**DOI:** 10.7554/eLife.02286

**Published:** 2014-05-23

**Authors:** Setsuko Wakao, Brian L Chin, Heidi K Ledford, Rachel M Dent, David Casero, Matteo Pellegrini, Sabeeha S Merchant, Krishna K Niyogi

**Affiliations:** 1 Department of Plant and Microbial Biology, University of California, Berkeley, Berkeley, United States; 2 Department of Molecular, Cell and Developmental Biology, University of California, Los Angeles, Los Angeles, United States; 3 Institute for Genomics and Proteomics, University of California, Los Angeles, Los Angeles, United States; 4 Department of Chemistry and Biochemistry, University of California, Los Angeles, Los Angeles, United States; 5 Howard Hughes Medical Institute, University of California, Berkeley, Berkeley, United States; 6 Physical Biosciences Division, Lawrence Berkeley National Laboratory, Berkeley, United States; Max Planck Institute for Developmental Biology, Germany

**Keywords:** chlamydomonas, singlet oxygen, retrograde signaling, photosynthesis, photo-oxidative stress, other

## Abstract

Singlet oxygen is a highly toxic and inevitable byproduct of oxygenic photosynthesis. The unicellular green alga *Chlamydomonas reinhardtii* is capable of acclimating specifically to singlet oxygen stress, but the retrograde signaling pathway from the chloroplast to the nucleus mediating this response is unknown. Here we describe a mutant, *singlet oxygen acclimation knocked-out 1* (*sak1*), that lacks the acclimation response to singlet oxygen. Analysis of genome-wide changes in RNA abundance during acclimation to singlet oxygen revealed that SAK1 is a key regulator of the gene expression response during acclimation. The *SAK1* gene encodes an uncharacterized protein with a domain conserved among chlorophytes and present in some bZIP transcription factors. The SAK1 protein is located in the cytosol, and it is induced and phosphorylated upon exposure to singlet oxygen, suggesting that it is a critical intermediate component of the retrograde signal transduction pathway leading to singlet oxygen acclimation.

**DOI:** http://dx.doi.org/10.7554/eLife.02286.001

## eLife digest

Plants, algae and some bacteria use photosynthesis to extract energy from sunlight and to convert carbon dioxide into the sugars needed for growth. One by-product of photosynthesis is a highly toxic molecule called singlet oxygen. Typically, organisms deal with stressful events such as the presence of toxic molecules by producing new proteins. However, protein production is generally initiated in the nucleus of the cell, and photosynthesis is carried out in structures called chloroplasts. Cells must therefore be able to alert the nucleus to the presence of toxic levels of singlet oxygen in the chloroplasts.

Like some plants that can withstand a gradual decrease in temperature, but not a sudden cold snap, the alga *Chlamydomonas reinhardtii* is capable of resisting high doses of singlet oxygen if it has previously been exposed to low doses of the molecule. Wakao et al. exploited this ability to hunt for algae that are unable to acclimate to singlet oxygen, and found that these cells are unable to produce a protein called SAK1.

Wakao et al. reveal that many factors involved in the algae's cellular response to singlet oxygen depend on the presence of SAK1. In addition, the response of the algae cells to singlet oxygen differs to the one seen in the model plant *Arabidopsis thaliana*, suggesting that the two organisms have found different ways to deal with the same problem.

The location of a protein in a cell can give clues to its function. SAK1 is present in the fluid surrounding cellular compartments—the cytosol—which is consistent with it acting as a signaling molecule between the chloroplast and the nucleus. Wakao et al. present further evidence for this hypothesis by demonstrating that the number of phosphate groups attached on SAK1 changes when exposed to singlet oxygen—a feature often seen in signaling proteins. In addition, part of SAK1 resembles proteins that can bind to DNA, which indicates that SAK1 may be directly involved in initiating protein production.

The discovery of SAK1 represents a starting point for understanding how the site of photosynthesis, the chloroplast, communicates with the nucleus. It also has implications for developing plants and algae that have a higher tolerance to environmental stress conditions for agriculture and biofuel production.

**DOI:** http://dx.doi.org/10.7554/eLife.02286.002

## Introduction

Growth of photosynthetic organisms depends on light energy, which in turn can cause oxidative damage to the cell if not managed properly (Li et al., 2009). Light intensity is highly dynamic in terrestrial and aquatic environments, and the cell must constantly control the dissipation of light energy to avoid photo-oxidative stress while maximizing productivity. In addition to being the site of photosynthesis, the chloroplast houses many essential biochemical reactions such as fatty acid and amino acid biosynthesis, but most of its proteins are encoded in the nucleus and must be imported after translation. Therefore the nucleus must monitor the status of the chloroplast and coordinate gene expression and synthesis of proteins to maintain healthy chloroplast functions.

It is known that signals originating from a stressed or dysfunctional chloroplast modulate nuclear gene expression, a process that is called retrograde signaling (Nott et al., 2006; Chi et al., 2013). In *Arabidopsis thaliana* the *gun* mutants have helped to define the field of chloroplast retrograde signaling, leading to the identification of GUN1, a pentatricopeptide repeat protein that is a regulator of this process (Koussevitzky et al., 2007), and pointing to the involvement of the tetrapyrrole biosynthetic pathway (Vinti et al., 2000; Mochizuki et al., 2001; Larkin et al., 2003; Strand et al., 2003; Woodson and Chory, 2008). A role for heme in retrograde signaling has been shown in *Chlamydomonas reinhardtii* as well (von Gromoff et al., 2008). Many of the *gun* studies were conducted in context of a dysfunctional chloroplast treated with norflurazon, an inhibitor of carotenoid biosynthesis. More recently a number of exciting advances have shed light on small molecules playing roles in retrograde stress signaling, including methylerythritol cyclodiphosphate, an intermediate of isoprenoid biosynthesis in the chloroplast (Xiao et al., 2012), 3-phosphoadenosine 5-phosphate (PAP) (Estavillo et al., 2011), as well as a chloroplast envelope transcription factor PTM (Sun et al., 2011). Plastid gene expression involving sigma factors has been implicated in affecting nuclear gene expression, although the mechanism is unknown (Coll et al., 2009; Woodson et al., 2012).

Activation of gene expression by reactive oxygen species (ROS) has been well documented (Apel and Hirt, 2004; Mittler et al., 2004; Gadjev et al., 2006; Li et al., 2009). Thus ROS have been proposed as a means for chloroplasts to signal stress to the nucleus and many examples of global gene expression changes in response to ROS have been described (Desikan et al., 2001; Vandenabeele et al., 2004; Vanderauwera et al., 2005). Singlet oxygen (^1^O_2_) is a highly toxic form of ROS that can be formed in all aerobic organisms through photosensitization reactions in which excitation energy is transferred from a pigment molecule to O_2_. For example, porphyria in humans is caused by defects in tetrapyrrole metabolism that can lead to accumulation of photosensitizing intermediates, which generate ^1^O_2_ in the light (Straka et al., 1990). In oxygenic photosynthetic organisms, ^1^O_2_ is mainly generated at the reaction center of photosystem II, when triplet excited chlorophyll transfers energy to O_2_ (Krieger-Liszkay, 2005). ^1^O_2_ is the predominant cause of lipid oxidation during photo-oxidative stress (Triantaphylidès et al., 2008) and is associated with damage to the reaction center (Trebst et al., 2002). Because of the abundance and proximity of the two elements of ^1^O_2_ generation, the photosensitizer chlorophyll and O_2_, it was hypothesized that oxygenic photosynthetic organisms must have evolved robust means to cope with this ROS (Knox and Dodge, 1985). In *Arabidopsis*, the EX1 and EX2 proteins in the chloroplast are required for the execution of a ^1^O_2_-dependent response: growth arrest in plants and programmed cell death in seedlings, that is distinct from cell damage (op den Camp et al., 2003; Wagner et al., 2004; Lee et al., 2007). Different players in ^1^O_2_ signaling have emerged recently, such as β-cyclocitral, an oxidation product of β-carotene in *Arabidopsis* (Ramel et al., 2012), a bZIP transcription factor (SOR1) responding to reactive electrophiles generated by ^1^O_2_ (Fischer et al., 2012), and a cytosolic zinc finger protein conserved in *Arabidopsis* and *Chlamydomonas,* MBS (Shao et al., 2013). In the anoxygenic photosynthetic bacterium *Rhodobacter sphaeroides*, a σ^E^ factor is responsible for the elicitation of the gene expression response to ^1^O_2_ (Anthony et al., 2005).

The unicellular green alga *Chlamydomonas reinhardtii* is an excellent model organism for investigation of retrograde ^1^O_2_ signaling. *Chlamydomonas* exhibits an acclimation response to ^1^O_2_, in which exposure to a sublethal dose of ^1^O_2_ leads to changes in nuclear gene expression that enable cells to resist a subsequent challenge with higher levels of ^1^O_2_ (Ledford et al., 2007). We hypothesized that acclimation mutants should include regulatory mutants that are defective in sensing and responding to ^1^O_2_. Here we describe the isolation of such a mutant and identification of a cytosolic phosphoprotein SAK1 that is critical for the acclimation and transcriptome response to ^1^O_2_.

## Results

### Isolation of a singlet oxygen-sensitive mutant that is defective in acclimation

**Figure 1.**
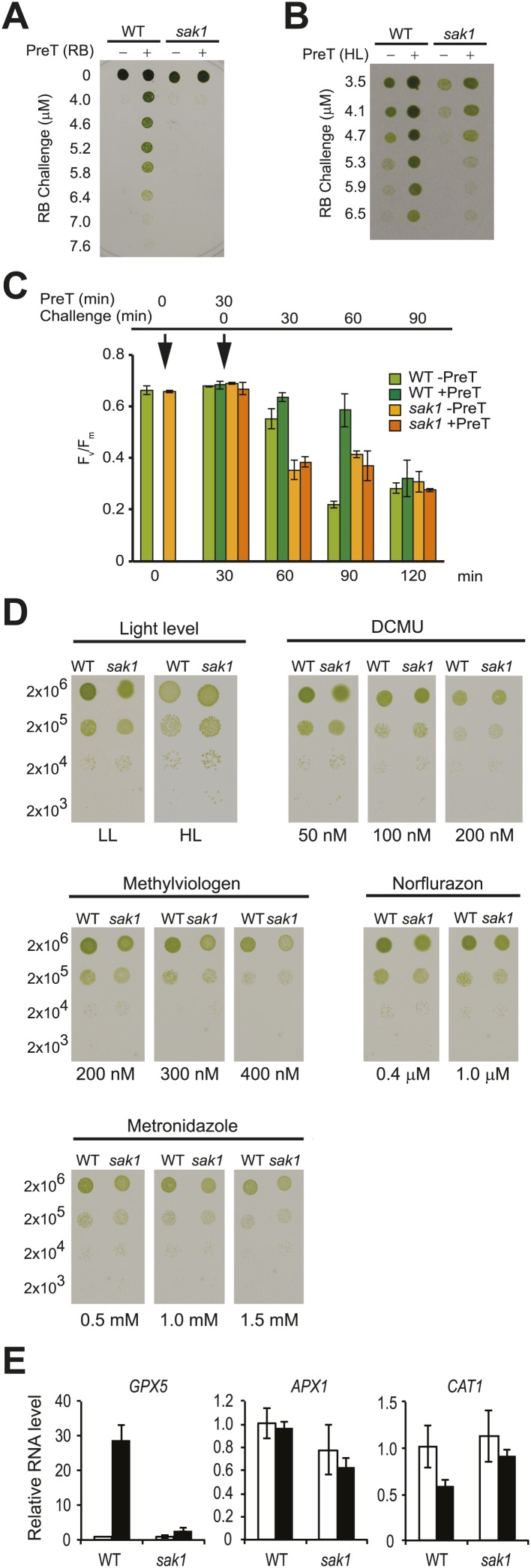
The *sak1* mutant is defective in singlet oxygen acclimation. (**A**) Acclimation phenotype of WT and *sak1*. The cells were pretreated in the dark (−) or under light (+) in the presence of rose bengal (RB), which requires light for generation of ^1^O_2_. Pretreatment was followed by a subsequent higher concentration of RB (Challenge) as indicated under light. (**B**) Cells grown in low light were either kept in low light (−) or transferred to high light (+) for an hour before challenge in the light with increasing RB concentrations. (**C**) F_v_/F_m_ values were measured after each time point indicated. Pretreatment (PreT) with 0.5 μM RB was applied for 30 min with (+PreT) or without (−PreT) light. After the pretreatment, RB was added to both dark and light samples to a final concentration of 3.75 μM RB (challenge), and F_v_/F_m_ was measured for 90 min at 30 min intervals (total 120 min). First arrow: addition of pretreatment; second arrow: addition of challenge. (**D**) *sak1* has wild-type sensitivity to other photo-oxidative stresses. Serial dilutions of WT and *sak1* were spotted onto minimal (HS) plates at the indicated light intensity or on TAP plates containing the indicated inhibitor. DCMU, 3-(3,4-dichlorophenyl)-1,1-dimethylurea; low light (LL), 80 µmol photons m^−2^ s^−1^; high light (HL), 450 µmol photons m^−2^ s^−1^. (**E**) Gene expression of a known ^1^O_2_-responsive gene, *GPX5,* is induced during acclimation, while two genes associated with H_2_O_2_ response, *APX1* and *CAT1,* are not. WT cells were mock-pretreated without RB (white bars) or pretreated with RB in the light (black bars). **DOI:** http://dx.doi.org/10.7554/eLife.02286.003

*Chlamydomonas* acclimates to singlet oxygen (^1^O_2_) generated by the exogenous photosensitizing dye rose bengal (RB) in the light (Ledford et al., 2007). As shown in Figure 1A, wild-type (WT) cells that were pretreated with RB in the light were able to survive a challenge treatment with much higher concentrations of RB, unlike cells pretreated with RB in the dark. By screening an insertional mutant population (Dent et al., 2005) for strains that were sensitive to ^1^O_2_, we isolated a mutant called *singlet oxygen acclimation knocked-out1* (*sak1*) that is defective in acclimation to ^1^O_2_ (Figure 1A). We have previously shown that *Chlamydomonas* WT cells can also acclimate to RB following pretreatment with high light (Ledford et al., 2007), indicating that high light and RB induce overlapping responses to ^1^O_2_. When subjected to the same conditions (high light pretreatment followed by challenge with RB), *sak1* demonstrated less robust cross-acclimation (Figure 1B). We also tested conversely whether pretreatment with RB can acclimate the cells to growth in high light or in the presence of norflurazon. No increase in resistance to high light or norflurazon was induced by pretreatment with RB in either WT or *sak1* (Figure 1—figure supplement 1). The viability phenotypes after RB treatment shown in Figure 1A were paralleled by changes in F_v_/F_m_ values, a chlorophyll fluorescence parameter representing photosystem II efficiency (Figure 1C). In both WT and *sak1,* pretreatment did not cause an inhibition of photosystem II, as demonstrated by unchanged F_v_/F_m_ values after 30 min. However, pretreatment increased resistance of photosystem II to the RB challenge only in WT and not in *sak1* cells (Figure 1C). The pretreatment protected the cells only transiently, as by 90 min of challenge treatment both genotypes appeared to have experienced similar inhibition of photosystem II (Figure 1C), consistent with the hypothesis that *sak1* is disrupted in early sensing and/or initiation of ^1^O_2_ response rather than its direct detoxification.

**Figure 1—figure supplement 1.**
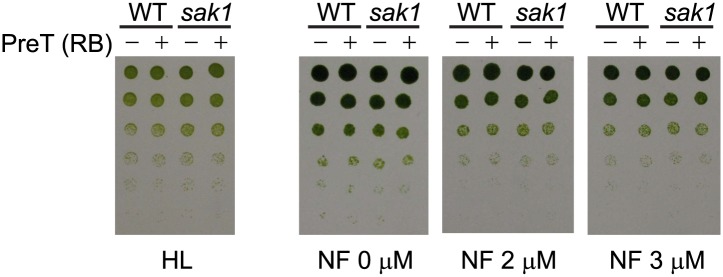
Pretreatment with RB does not increase resistance to high light or norflurazon in cells grown on plates. Cells were pretreated with 1 μM RB with (+) or without (−) light, then spotted on minimal plates and grown under high light (HL) or grown photoheterotrophically on TAP plates containing norflurazon (NF) and grown under low light for 4 days. Cells were spotted in serial dilutions. **DOI:** http://dx.doi.org/10.7554/eLife.02286.004

In contrast to its RB sensitivity, *sak1* exhibited wild-type resistance to high light, various photosynthetic inhibitors and generators of other ROS, suggesting its defect is specific to ^1^O_2_ (Figure 1D). When tested for the gene expression response of the known ^1^O_2_-specific gene *GPX5* (Leisinger et al., 2001) during acclimation, WT cells showed a 20- to 30-fold induction, whereas a known H_2_O_2_-responsive ascorbate peroxidase gene (*APX1*) in *Chlamydomonas* (Urzica et al., 2012) and a catalase gene (*CAT1*), known to be H_2_O_2_ responsive in *Arabidopsis* (Davletova et al., 2005; Vanderauwera et al., 2005), were unchanged. The mutant *sak1* showed attenuated *GPX5* induction, as expected for a mutant defective in the ^1^O_2_ response (Figure 1E).

### The global gene expression response to ^1^O_2_ in *Chlamydomonas* is distinct from that in *Arabidopsis*

**Figure 2.**
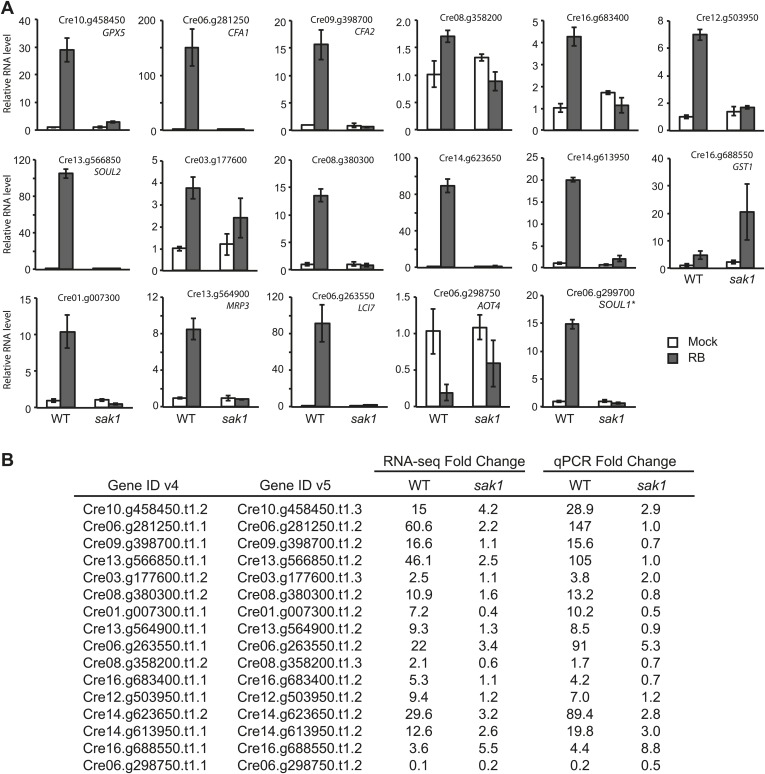
qRT-PCR analysis of genes identified to be ^1^O_2_-responsive by RNA-seq. (**A**) The error bars indicate standard deviation of biological triplicates. The locus of the transcript (v5) and gene name if annotated, are indicated. *SOUL1 was named gene in v4 but not in v5. (**B**) Comparison of fold change values from RNA-seq data and qPCR. Fold change values were calculated for RNA-seq as described in ‘Material and methods’, and the values for qPCR are averages obtained from biological triplicates. **DOI:** http://dx.doi.org/10.7554/eLife.02286.005

To obtain insight into the cellular processes and the genes involved in ^1^O_2_ acclimation, we used RNA-seq to define the transcriptome of WT cells during acclimation. The sequences were mapped to the *Chlamydomonas reinhardtii* genome version 4 (v4), and 16476 transcripts corresponding to gene models were detected (Wakao et al., 2014). We validated the data by quantitative reverse transcriptase PCR (qRT-PCR) for some of the differentially expressed genes during acclimation (Figure 2). Basal expression of some of the genes was elevated in *sak1* compared to WT (Cre16.g683400 and *GST1,* Figure 2). Comparisons of the fold change (FC) values obtained by RNA-seq and qRT-PCR for the genes tested in Figure 2 are shown in Figure 2. The FC values are comparable between the two methods, although genes with FC greater than 20 (detected by RNA-seq) showed FC values (estimated by qRT-PCR) that were two to three times higher (Cre06.g281250.t1.1, Cre13.g566850.t1.1, Cre06.g263550.t1.1, Cre14.g623650.t1.2). Some of the genes were also induced by a transition from low light to high light, although not as strongly (Table 1), indicating that the ^1^O_2_ response elicited by addition of RB partly overlaps with that caused by increased light intensity. To examine whether the transcriptome changes were specific to ^1^O_2_, we examined the expression of several previously identified H_2_O_2_-responsive genes (Urzica et al., 2012) (Table 2). Two of the seven genes, *VTC2* (3.4-fold) and *DHAR1* (twofold) were induced during ^1^O_2_ acclimation, whereas the other five genes were not differentially expressed (induced more than twofold) in our data. For these two genes, their magnitude of induction by ^1^O_2_ was smaller than that of H_2_O_2_-treated cells (both genes were ∼ninefold induced by 1 mM H_2_O_2_ treatment for 60 min) (Urzica et al., 2012). These differences suggest that our treatment with ^1^O_2_ did not lead to a large-scale induction of H_2_O_2_-responsive genes, and it is likely that the two above-mentioned genes involved in ascorbate metabolism respond to both H_2_O_2_ and ^1^O_2_.

**Table 1.** Moderate induction of ^1^O_2_ genes during high light exposure **DOI:** http://dx.doi.org/10.7554/eLife.02286.006

	Fold change (SD)*	
Gene name or ID	WT	*sak1*
*GPX5*	2.86 (1.06)	1.08 (0.23)
*CFA1*	3.75 (0.99)	1.78 (0.52)
*SOUL2*	3.45 (1.25)	1.82 (0.22)
*MRP3*	3.10 (0.39)	2.37 (0.32)
Cre14.g613950	1.42 (0.53)	1.57 (0.46)
*LHCSR1*†	14.91 (4.25)	2.91 (1.35)

* Fold change values are the average of biological triplicates and their standard deviations are indicated in parentheses.

† Known to have elevated expression in high light grown cells (Peers et al., 2009).

**Table 2.** Expression of H_2_O_2_ response genes during ^1^O_2_ acclimation **DOI:** http://dx.doi.org/10.7554/eLife.02286.007

	Gene ID		RPKM*				Fold change†	
Gene name	v4	v5	WT-mock	WT-RB	*sak1*-mock	*sak1*-RB	WT	*sak1*
*APX1*	Cre02.g087700.t1.1	Cre02.g087700.t1.2	49.70	36.22	79.65	58.83	0.73	0.74
*MSD3*	Cre16.g676150.t1.1	Cre16.g676150.t1.2	0.30	0.18	0.70	0.17	0.60	0.25
*MDAR1*	Cre17.g712100.t1.1	Cre17.g712100.t1.2	35.95	38.30	33.53	51.34	1.07	1.53
*DHAR1*	Cre10.g456750.t1.1	Cre10.g456750.t1.2	20.40	40.93	25.69	42.18	2.01	1.64
*GSH1*	Cre02.g077100.t1.1	Cre02.g077100.t1.2	28.27	26.91	40.42	49.95	0.95	1.24
*GSHR1*	Cre06.g262100.t1.2	Cre06.g262100.t1.3	19.17	19.02	19.39	22.41	0.99	1.16
*VTC2*	Cre13.g588150.t1.1	Cre13.g588150.t1.2	18.16	62.53	35.10	103.12	3.44	2.94

* Average of RPKM obtained from two sequencing lanes as described in ‘Material and methods’.

† Calculated as ratio of (RPKM-RB) / (RPKM-mock).

**Figure 3.**
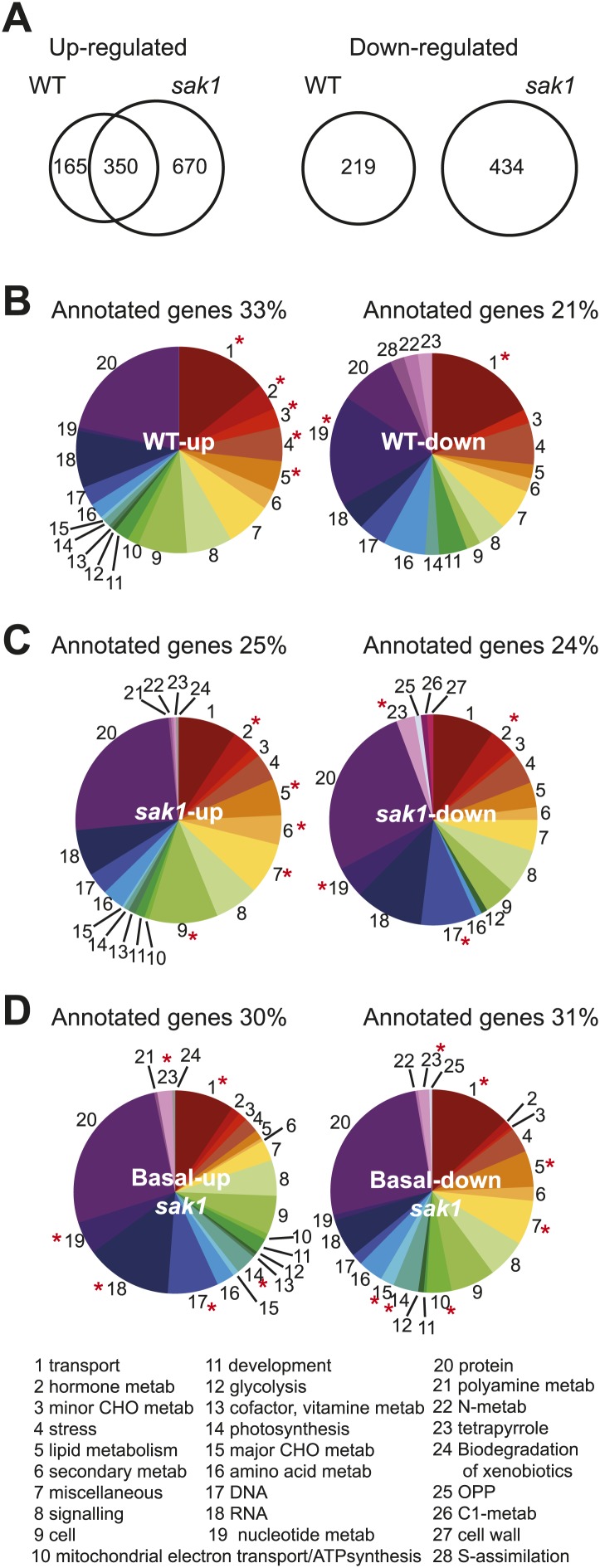
Differentially expressed genes from pair-wise comparisons. (**A**) Venn diagram representing differentially expressed genes in WT and *sak1.* Mapman functional classes distribution of differentially expressed genes (passing criteria of fold change greater than 2^1^ [up] or smaller than 2^−1^ [down] with FDR <1%) during acclimation in (**B**) WT and (**C**) *sak1.* (**D**) Differentially expressed genes when comparing WT and *sak1* in basal conditions (i.e., before exposure to ^1^O_2_). The functional classes represented by the numbers are listed; asterisks indicate classes that were enriched compared to the genome. **DOI:** http://dx.doi.org/10.7554/eLife.02286.008

During acclimation of WT to ^1^O_2_, 515 genes were up-regulated at least twofold with a false discovery rate (FDR) smaller than 1% (Supplementary file 1, C1), and 33% of these could be categorized into functional classes based on MapMan (Thimm et al., 2004) using the Algal Functional Annotation Tool (Lopez et al., 2011) (Figure 3A,B). The enriched classes are marked with asterisks, and the genes within those classes are listed in Table 3. Genes involved in sterol/squalene/brassinosteroid metabolism (in the hormone and lipid metabolism functional classes) were notably enriched (Table 3). A sterol methyltransferase was also detected to display differential expression in our previous microarray analysis (Ledford et al., 2007). Brassinosteroids are not known to exist in *Chlamydomonas*, and in plants increasing evidence indicates sterols have a signaling role independent of brassinosteroids (Lindsey et al., 2003; Boutté and Grebe, 2009). Two cyclopropane fatty acid synthases (CFAs) were among the up-regulated lipid metabolism genes (Table 3). Another function that was notable among up-regulated genes, although they were not grouped to a common functional class by MapMan, were two genes coding for SOUL heme-binding domain proteins that were *SAK1*-dependent (SOUL2 and Cre06.g299700.t1.1, formerly annotated as SOUL1) (Figure 2). Genes annotated as involved in transport comprised one of the most enriched classes (Figure 3B). These included a number of multidrug-resistant (MDR) and pleiotropic drug-resistant (PDR) type transporters as well as other various transporters for ions, peptides, and lipids (Table 3). The former types of transporters may reflect the cells' response to pump RB out. When the responses to the chemical RB and ^1^O_2_ were uncoupled by comparing gene expression in cultures kept in the dark with and without RB, all of the tested ^1^O_2_-induced genes and ABC transporters identified from our RNA-seq remained unchanged by RB in the dark in both WT and *sak1* (Table 4). This result indicates that the up-regulation of these genes when RB was added in the light was a response to ^1^O_2_ rather than to RB itself. Up-regulation of stress genes included those coding for chaperones and some receptor-like proteins (Figure 3B; Table 3), suggesting that the cells do mount a stress response during acclimation though not visible by gross growth phenotype (Figure 1A) or decrease in F_v_/F_m_ (Figure 1C). A smaller number of 219 genes was down-regulated during acclimation in WT (Supplementary file 1, C1), only 21% of which had functional annotation. The most enriched classes of down-regulated genes were nucleotide metabolism and transport, the latter including a distinct type of transporter for small metabolites and ions, different from those found among up-regulated genes that included many MDR- and PDR-type transporters (Figure 3B; Table 3).

**Table 3.** Enriched functional classes among differentially expressed genes in WT during ^1^O_2_ acclimation **DOI:** http://dx.doi.org/10.7554/eLife.02286.009

Primary MapMan class	Secondary Mapman class	Gene ID (v4)	Gene ID (v5)	Gene name	Annotation
Up-regulated genes					
transport	ABC transporters and multidrug resistance systems	Cre03.g169300.t1.1	Cre03.g169300.t2.1		ABC transporter (ABC-2 type)
		Cre04.g220850.t1.1	Cre04.g220850.t1.2		ABC transporter (ABC-2 type)
		Cre11.g474600.t1.1§	Cre02.g095151.t1		ABC transporter (ABC-2 type)
		Cre03.g151400.t1.2	Cre03.g151400.t1.3		ABC transporter (subfamilyA member3)
		Cre14.g618400.t1.1§	Cre14.g618400.t1.2		ABC transporter
		Cre09.g395750.t1.2	Cre09.g395750.t1.3		ABC transporter (plant PDR pleitropic drug resistance)
		Cre14.g613950.t1.1§	Cre14.g613950.t2.1		ABC transporter, Lipid exporter ABCA1 and related proteins
		Cre17.g725150.t1.1	Cre17.g725150.t1.2		ABC transporter
		Cre04.g224400.t1.2§	Cre04.g224400.t1.3		ABC transporter (plant PDR pleitropic drug resistance)
		Cre13.g564900.t1.1§	Cre13.g564900.t1.2	*MRP3*	ABC transporter, Multidrug resistance associated protein
		Cre17.g721000.t1.1	Cre17.g721000.t1.2		ABC transporter (ABCA)
		Cre04.g224500.t1.2	Cre04.g224500.t1.3		ABC transporter (plant PDR pleitropic drug resistance)
		Cre01.g007000.t1.1§	Cre01.g007000.t1.2		ABC transporter (ABC-2 type)
	unspecified anions	Cre13.g574000.t1.2	Cre13.g574000.t1.3		Chloride channel 7
		Cre17.g729450.t1.1	Cre17.g729450.t1.2		Chloride channel 7
	amino acids	Cre04.g226150.t1.2	Cre04.g226150.t1.3	*AOC1*	Amino acid carrier 1; belongs to APC (amino acid polyamine organocation) family
	misc	Cre16.g683400.t1.1§	Cre16.g683400.t1.2		CRAL/TRIO domain (Retinaldehyde binding protein-related)
		Cre17.g718100.t1.1	Cre17.g718100.t1.2		Phosphatidylinositol transfer protein SEC14 and related proteins (CRAL/TRIO)
		Cre06.g311000.t1.2	Cre06.g311000.t1.3	*FBT2*	Folate transporte
	calcium	Cre09.g410050.t1.1§	Cre09.g410050.t1.2		Ca2+ transporting ATPase
	potassium	Cre07.g329882.t1.2	Cre07.g329882.t1.3		Ca2+-activated K+ channel proteins
	phosphate	Cre16.g686750.t1.1	Cre16.g686750.t1.2	*PTA3*	Proton/phosphate symporter
	metal	Cre13.g570600.t1.1	Cre13.g570600.t1.2	*CTR1*	CTR type copper ion transporter
	metabolite transporters at the mitochondrial membrane	Cre06.g267800.t1.2	Cre06.g267800.t2.1		Mitochondrial carrier protein
hormone metabolism*	brassinosteroid	Cre16.g663950.t1.1	Cre16.g663950.t1.2		Sterol C5-desaturase
		Cre02.g076800.t1.1	Cre02.g076800.t1.2		delta14-sterol reductase
		Cre12.g557900.t1.1	Cre12.g557900.t1.1	*CDI1*	C-8,7 sterol isomerase
		Cre02.g092350.t1.1	Cre02.g092350.t1.2		Cytochrome P450, CYP51 Sterol-demethylase
		Cre12.g500500.t1.2	Cre12.g500500.t2.1		SAM-dependent methyltransferases
	jasmonate	Cre19.g756100.t1.1	Cre03.g210513.t1		12-oxophytodienoic acid reductase
	auxin	Cre14.g609900.t1.1	Cre14.g609900.t1.1		Predicted membrane protein, contains DoH and Cytochrome b-561/ferric reductase transmembrane domains
		Cre06.g276050.t1.1	Cre06.g276050.t1.2		Aldo/keto reductase
		Cre16.g692800.t1.2	Cre16.g692800.t1.3		Aldo/keto reductase
		Cre03.g185850.t1.2	Cre03.g185850.t1.2		pfkB family, sugar kinase-related
minor CHO metabolism	others	Cre06.g276050.t1.1	Cre06.g276050.t1.2		Aldo/keto reductase
		Cre16.g692800.t1.2	Cre16.g692800.t1.3		Aldo/keto reductase
		Cre03.g185850.t1.2	Cre03.g185850.t1.2		pfkB family, sugar kinase-related
	callose	Cre06.g302050.t1.1	Cre06.g302050.t1.2		1,3-beta-glucan synthase
	myo-inositol	Cre03.g180250.t1.1	Cre03.g180250.t1.2		Myo-inositol-1-phosphate synthase
stress	biotic	Cre01.g057050.t1.1§	Cre03.g144324.t1		Leucine Rich Repeat
		Cre01.g016200.t1.2	Cre01.g016200.t1		Mlo Family
		Cre28.g776450.t1.1§	Cre08.g358573.t1	*PSMD10*	26S proteasome regulatory complex
	abiotic	Cre12.g501500.t1.1	NF†		
		Cre02.g132300.t1.2	Cre09.g395732.t1		DnaJ domain
		Cre07.g339650.t1.2	Cre07.g339650.t1.3	*DNJ20*	DnaJ-like protein
		Cre01.g033300.t1.1§	Cre01.g033300.t2.1		No annotation‡
		Cre16.g677000.t1.1	Cre16.g677000.t1.2	*HSP70E*	Heat shock protein 70E
		Cre08.g372100.t1.1	Cre08.g372100.t1.2	*HSP70A*	Heat shock protein 70A
lipid metabolism	phospholipid synthesis	Cre13.g604700.t1.2	Cre13.g604700.t1.3	*PCT1*	CDP-alcohol phosphatidyltransferase/Phosphatidylglycerol-phosphate synthase
		Cre06.g281250.t1.1§	Cre06.g281250.t1.2	*CFA1*	Cyclopropane fatty acid synthase
		Cre09.g398700.t1.1§	Cre09.g398700.t1.2	*CFA2*	Cyclopropane fatty acid synthase
	‘exoticsߣ (steroids, squalene etc)	Cre01.g061750.t1.1	Cre03.g146507.t1	*SPT2*	Serine palmitoyltransferase
		Cre83.g796250.t1.1	NF†	*SPT1*	Serine palmitoyltransferase
		Cre02.g137850.t1.1	Cre09.g400516.t1		TRAM (translocating chain-associating membrane) superfamily
	FA synthesis and FA elongation	Cre03.g182050.t1.1	Cre03.g182050.t1		Long-chain acyl-CoA synthetases (AMP-forming)
		Cre06.g256750.t1.1	Cre06.g256750.t1.2		Acyl-ACP thioesterase
misc	short chain dehydrogenase/reductase (SDR)	Cre12.g556750.t1.2	Cre12.g556750.t1.3		Short chain dehydrogenase
		Cre27.g775000.t1.1	Cre12.g549852.t1		Short chain dehydrogenase
		Cre17.g731350.t1.2	Cre17.g731350.t1.2		Short chain dehydrogenase
		Cre08.g381510.t1.1§	NF†		Short chain alcohol dehydrogenase
	UDP glucosyl and glucoronyl transferases	Cre02.g144050.t1.1	Cre02.g144050.t2.1		Acetylglucosaminyltransferase EXT1/exostosin 1
		Cre16.g659450.t1.1	Cre16.g659450.t1.2		Lactosylceramide 4-alpha-Galactosyltransferase
		Cre03.g173300.t1.1	Cre03.g173300.t1.2		Lactosylceramide 4-alpha-Galactosyltransferase
	dynamin	Cre02.g079550.t1.1	Cre02.g079550.t1.2		Dynamin-related GTPase, involved in circadian rhythms
	misc2	Cre06.g258600.t1.1§	Cre06.g258600.t2.1		Predicted hydrolase related to dienelactone hydrolase
	acid and other phosphatases	Cre06.g249800.t1.1	Cre06.g249800.t1.2		Sphingomyelin synthase
Down-regulated genes					
nucleotide metabolism	salvage	Cre13.g573800.t1.1	Cre13.g573800.t1.2		Phosphoribulokinase / Uridine kinase family
	synthesis	Cre12.g503300.t1.1	Cre12.g503300.t1.2		Phosphoribosylamidoimidazole-succinocarboxamide synthase
		Cre06.g308500.t1.1	Cre06.g308500.t1.2	*CMP2*	Carbamoyl phosphate synthase, small subunit
		Cre14.g614300.t1.1	Cre14.g614300.t1.2		Inosine-5-monophosphate dehydrogenase
transport	ABC transporters and multidrug resistance systems	Cre06.g273750.t1.2	Cre06.g273750.t1.3	*SUA1*	Chloroplast sulfate transporter
		Cre02.g083354.t1.1	Cre02.g083354.t1		ATP-binding cassette, subfamily B (MDR/TAP), member 9
	calcium	Cre06.g263950.t1.2	Cre06.g263950.t1.3		Na+/K + ATPase, alpha subunit
	metabolite transporters at the envelope membrane	Cre08.g363600.t1.1	Cre08.g363600.t1.2		Glucose-6-phosphate, PEP/phosphate antiporter
	metal	Cre17.g720400.t1.2	Cre17.g720400.t1.3	*HMA1*	Heavy metal transporting ATPase
	P- and V-ATPases	Cre10.g459200.t1.1	Cre10.g459200.t1.2	*ACA4*	Plasma membrane H + -transporting ATPase
	phosphate	Cre02.g144650.t1.1	Cre02.g144650.t1.2	*PTB12*	Na+/Pi symporter
	potassium	Cre06.g278700.t1.2	Cre06.g278700.t1.2		Myotrophin and similar proteins

* Functional terms are inferred by homology to the annotation set of *Arabidopsis thaliana* (Lopez et al., 2011).

† Corresponding gene model was not found in v5.

‡ No functional annotations found on v5 but defined by MapMan on Algal Functional Annotation Tool (Lopez et al., 2011).

§ Induction during ^1^O_2_ acclimation dependent on SAK1 (Table 5).

**Table 4.** ^1^O_2_ response genes are not induced when RB is added in the dark **DOI:** http://dx.doi.org/10.7554/eLife.02286.010

	Fold change +RB/−RB (SD)*	
Gene name or ID	WT	*sak1*
*GPX5*	1.13 (0.33)	0.87 (0.31)
*SAK1*	1.38 (0.08)	1.29 (0.19)
*CFA1*	0.90 (0.04)	1.44 (0.22)
*SOUL2*	1.17 (0.25)	1.11 (0.19)
*MRP3*†,‡	1.13 (0.12)	1.07 (0.25)
Cre12.g503950†,‡	0.93 (0.06)	1.20 (0.12)
Cre14.g613950†,§	0.65 (0.06)	0.79 (0.15)
Cre04.g220850†,‡	1.00 (0.09)	1.29 (0.04)
Cre09.g395750†,‡	1.05 (0.10)	1.29 (0.12)

* Average of fold change and standard deviation (SD) of biological triplicates.

† Annotated as transport function.

‡ ABC transporter.

§ Sec14-like phosphatidylinositol transfer protein.

Although only 33% of the up-regulated genes have a functional annotation (Figure 3B), it is interesting that the ^1^O_2_ response in *Chlamydomonas* involves genes and biological processes that appear to be distinct from those that respond specifically to ^1^O_2_ in *Arabidopsis* (op den Camp et al., 2003). A total of 70 ^1^O_2_-response genes have been defined using a microarray with the *flu* mutant in *Arabidopsis* (op den Camp et al., 2003). These genes include the following classes (number of genes): metabolism (11), transcription (5), protein fate (4), transport (2), cellular communication/signal transduction (17), cell rescue/defense in virulence (4), subcellular localization (2), binding function or cofactor requirement (1), transport facilitation (5) and others (19). From this list of 70 genes we found four similarly annotated genes within our 515 genes induced by ^1^O_2_ in *Chlamydomonas*: a Myb transcription factor, a mitochondrial carrier protein, an amino acid permease, and an ATPase/aminophospholipid translocase. None of these genes in *Chlamydomonas* was the closest ortholog of the corresponding *Arabidopsis* gene*.* Conversely, genes similar to those strongly up-regulated in a SAK1-dependent manner such as CFAs, SOUL proteins, GPX, and sterol biosynthetic enzymes were not found among the *Arabidopsis* ^1^O_2_-specific genes despite having clear counterparts in *Arabidopsis*. Taken together, these results suggest that these two organisms may deploy distinct mechanisms in their responses to ^1^O_2_.

### The *sak1* mutant is defective in the global gene expression response during acclimation to ^1^O_2_

In the *sak1* mutant*,* 1020 genes were up-regulated, whereas 434 genes were down-regulated during acclimation (Supplementary file 1, C2). 350 of the 515 genes up-regulated in WT overlapped with the set of up-regulated genes in the mutant (Figure 3A). Comparing the fold changes of genes in WT and *sak1* during acclimation, we defined 104 genes as SAK1-dependent genes that displayed moderate to strong attenuation in their response (fold change ratio <0.5) (Table 5). Some of the genes that belong to enriched biological classes found among WT up-regulated genes are indicated in Table 3. Interestingly, the most strongly induced genes in WT were found among this group; 37 out of 104 SAK1-dependent genes were among the top 10% most strongly induced genes (Table 5). 33 out of these 37 most strongly induced SAK1-dependent genes displayed strong disruption in their up-regulation; reduced to 0.01–0.25 of magnitude of fold change in *sak1* as compared to WT (Table 5). These results indicate SAK1 is required for the induction of the most strongly induced genes during acclimation reflecting its critical role in regulating the cellular acclimation response to ^1^O_2_.

**Table 5.** Genes that require *SAK1* for induction by ^1^O_2_ **DOI:** http://dx.doi.org/10.7554/eLife.02286.011

Gene ID (v4)	Gene ID (v5)	Gene name	Annotation	FC WT* (log_2_)	FC *sak1* (log_2_)	Attenuation (FC-*sak1*/FC-WT)†	Basal repression in *sak1* (log_2_)
Cre02.g137700.t1.1‡	Cre09.g400404			6.49	1.80	0.04	−3.35
Cre06.g281250.t1.1‡	Cre06.g281250	*CFA1*	Cyclopropane fatty acid synthase	5.92	1.16	0.04	−2.10
Cre27.g775950.t1.2	Cre12.g557928			5.83	0.81	0.03	
Cre01.g033300.t1.1	Cre01.g033300			5.72	−0.39	0.01	
Cre13.g566850.t1.1‡	Cre13.g566850	*SOUL2*	SOUL heme-binding protein	5.53	1.33	0.05	−2.60
Cre14.g623650.t1.1	Cre14.g623650		Alcohol dehydrogenase	4.89	1.67	0.11	
Cre13.g600650.t1.1	Cre06.g278245		Rieske 2Fe-2S domain	4.76	1.64	0.12	
Cre06.g263550.t1.1	Cre06.g263550	*LCI7*	R53.5-related protein	4.46	1.77	0.15	
Cre07.g342100.t1.1	Cre07.g342100			4.43	1.40	0.12	
Cre06.g299700.t1.1‡	Cre06.g299700	*SOUL1*	SOUL heme-binding protein	4.32	0.43	0.07	−1.13
Cre09.g398700.t1.1‡	Cre09.g398700	*CFA2*	Cyclopropane fatty acid synthase	4.05	0.18	0.07	−1.00
Cre12.g492650.t1.1‡	Cre12.g492650	*FAS2*	Fasciclin-like protein	4.01	0.07	0.07	−1.24
Cre08.g381510.t1.1	NF			3.94	0.73	0.11	
Cre10.g458450.t1.2	Cre10.g458450	*GPX5*	Glutathione peroxidase	3.91	2.06	0.28	
Cre11.g474600.t1.1	Cre02.g095151		ABC transporter (ABC-2 type)	3.90	0.44	0.09	
Cre13.g600700.t1.1	Cre06.g278246			3.78	1.48	0.20	
Cre14.g613950.t1.1	Cre14.g613950			3.65	1.38	0.21	
Cre06.g269300.t1.1	Cre06.g269300		DUF1365	3.50	0.40	0.12	
Cre08.g380300.t1.2	Cre08.g380300	*MSRA3*	Peptide methionine sulfoxide reductase	3.45	0.66	0.14	
Cre28.g776450.t1.1	Cre08.g358573	*TRP7*	Transient receptor potential ion channel	3.31	−0.79	0.06	
Cre01.g031650.t1.2	Cre01.g031650	*CGLD12*	Potential galactosyl transferase activity	3.30	0.67	0.16	
Cre14.g629061.t1.1	NF		DUF2177	3.25	0.08	0.11	
Cre12.g503950.t1.1	Cre12.g503950		CRAL/TRIO domain	3.24	0.31	0.13	
Cre13.g564900.t1.1	Cre13.g564900		ABC transporter transmembrane region	3.22	0.34	0.14	
Cre02.g139500.t1.1	Cre09.g401701		DUF1295	3.04	−0.16	0.11	
Cre14.g618400.t1.1	Cre14.g618400			2.97	1.15	0.28	
Cre17.g715150.t1.1	Cre17.g715150			2.89	0.13	0.15	
Cre17.g741300.t1.2‡	Cre17.g741300	*SAK1*		2.88	0.66	0.21	−2.77
Cre01.g007300.t1.1	Cre01.g007300			2.85	−1.15	0.06	
Cre16.g648700.t1.2‡	Cre16.g648700		ABC transporter (ABC-2 type)	2.79	0.26	0.17	−1.26
Cre13.g566900.t1.2	Cre13.g566900			2.76	−0.38	0.11	
Cre02.g137750.t1.2	Cre09.g400441		JmjC domain	2.72	−0.31	0.12	
Cre06.g263500.t1.1	Cre06.g263500		Archease protein family (DUF101)	2.67	1.02	0.32	
Cre01.g016150.t1.1‡	Cre01.g016150		ADP-ribosylglycohydrolase	2.65	0.17	0.18	−1.26
Cre08.g380000.t1.1	Cre08.g380000		Formylglycine-generating sulfatase enzyme	2.59	1.53	0.48	
Cre14.g615600.t1.1	Cre14.g615600		Putative serine esterase (DUF676)	2.53	−0.54	0.12	
Cre11.g472900.t1.2	Cre02.g095113		CAP-Gly domain	2.45	−0.05	0.18	
Cre06.g269250.t1.1	Cre06.g269250			2.44	0.55	0.27	
Cre02.g120600.t1.1	Cre09.g403071			2.44	0.94	0.35	
Cre06.g261200.t1.1	Cre06.g261200	*ERG25*	Sterol desaturase	2.42	0.64	0.29	
Cre16.g683400.t1.1	Cre16.g683400		CRAL/TRIO domain	2.40	0.08	0.20	
Cre22.g765150.t1.1	Cre11.g467725		hypothetical protein	2.30	0.46	0.28	
Cre13.g571800.t1.2	Cre13.g571800		DUF1336	2.27	0.72	0.34	
Cre13.g579450.t1.2	Cre13.g579450	*CST1*	Membrane transporter	2.27	1.23	0.49	
Cre08.g380350.t1.1	Cre08.g380350			2.21	−0.01	0.21	
Cre16.g649250.t1.2	Cre16.g649250			2.08	0.58	0.35	
Cre11.g476250.t1.1	Cre11.g476250			2.08	0.49	0.33	
Cre02.g108000.t1.2	Cre02.g108000			2.08	1.03	0.49	
Cre13.g583300.t1.1	Cre13.g583300			1.98	−0.48	0.18	
Cre04.g215300.t1.2	NF			1.97	0.57	0.38	
Cre02.g139450.t1.1	Cre09.g401663		DUF947	1.95	−0.62	0.17	
Cre03.g194750.t1.2	Cre03.g194750			1.95	0.73	0.43	
Cre06.g258600.t1.1	Cre06.g258600		Dienelactone hydrolase family	1.91	−0.95	0.14	
Cre10.g418700.t1.1	Cre10.g418700		Probable N6-adenine methyltransferase	1.87	−0.03	0.27	
Cre10.g444550.t1.1	Cre10.g444550	*SPP1A*	Signal peptide peptidase	1.81	0.51	0.41	
Cre01.g060050.t1.2	Cre03.g145807			1.78	−0.11	0.27	
Cre09.g410050.t1.1	Cre09.g410050		Calcium transporting ATPase	1.76	0.51	0.42	
Cre03.g163400.t1.2	Cre03.g163400			1.76	−0.17	0.26	
Cre01.g008450.t1.1	Cre01.g008450		Nuf2 family	1.73	−0.54	0.21	
Cre12.g536650.t1.1	Cre12.g536650			1.72	0.35	0.39	
Cre02.g114900.t1.2	Cre02.g114900	*ANK23*	predicted protein	1.71	0.08	0.32	
Cre16.g661850.t1.2	Cre16.g661850		Calcium/calmoduline dependent protein kinase association	1.69	0.03	0.32	
Cre14.g615500.t1.2	Cre14.g615500		Glycoprotease family	1.68	−0.76	0.18	
Cre11.g483100.t1.2	Cre11.g483100		Protein kinase	1.66	−0.49	0.22	
Cre28.g776650.t1.1	Cre08.g358569			1.64	0.33	0.40	
Cre07.g340250.t1.2	Cre07.g340250		Protein kinase	1.63	−0.41	0.24	
Cre06.g296250.t1.2	Cre06.g296250	*SYK1*	tRNA synthetase, class II	1.60	0.54	0.48	
Cre06.g310500.t1.1	Cre06.g310500			1.57	0.18	0.38	
Cre07.g342800.t1.2	Cre07.g342800	*CGL16*	Predicted protein	1.49	0.32	0.44	
Cre03.g181450.t1.2	Cre03.g181450		DUF1619	1.47	0.35	0.46	
Cre66.g793601.t1.1	Cre35.g759497			1.47	0.03	0.37	
Cre14.g614050.t1.2	Cre14.g614050	*MAP65*	Microtubule associated protein	1.43	0.06	0.39	
Cre04.g217500.t1.1	Cre04.g217500		Inosine-uridine preferring nucleoside hydrolase	1.42	0.19	0.43	
Cre06.g292950.t1.1	Cre06.g292950		DNA polymerase delta, subunit 4	1.38	−0.12	0.35	
Cre16.g661750.t1.1	Cre16.g661750		Calcium/calmoduline dependent protein kinase association	1.38	−0.12	0.35	
Cre01.g007000.t1.1	Cre01.g007000		ABC transporter (ABC-2 type)	1.35	0.21	0.45	
Cre04.g224400.t1.2	Cre04.g224400		ABC transporter (ABC-2 type)	1.34	−0.13	0.36	
Cre01.g068400.t1.2	Cre16.g680790			1.33	0.16	0.45	
Cre05.g237400.t1.1	Cre05.g237400	*DAE1*	Diaminopimelate epimerase	1.32	0.22	0.47	
Cre14.g609600.t1.2	Cre14.g609600			1.32	−0.58	0.27	
Cre05.g234850.t1.2	Cre05.g234850		Ubiquitin carboxyl-terminal hydrolase	1.29	0.16	0.46	
Cre03.g179200.t1.1	Cre03.g179200			1.28	−0.48	0.30	
Cre10.g417730.t1.1	Cre10.g417730			1.27	0.17	0.47	
Cre03.g159700.t1.2	Cre03.g159700			1.26	−0.14	0.38	
Cre12.g540150.t1.2	Cre12.g540150			1.19	−0.24	0.37	
Cre01.g006550.t1.2‡	Cre01.g006550		No annotation	1.17	−0.49	0.32	−1.60
Cre03.g159950.t1.2	Cre03.g159950			1.17	−0.17	0.40	
Cre27.g775900.t1.2	Cre12.g557503			1.14	−0.70	0.28	
Cre02.g121600.t1.1	Cre09.g387208		Protein kinase	1.14	0.00	0.46	
Cre14.g609550.t1.1	NF			1.13	−0.84	0.26	
Cre07.g315050.t1.2	Cre07.g315050			1.12	−0.03	0.45	
Cre04.g218800.t1.2	Cre04.g218800	*THB3*	Truncated hemoglobin	1.11	−0.50	0.33	
Cre02.g133300.t1.1	Cre09.g396624			1.11	−0.43	0.34	
Cre01.g060650.t1.2	Cre03.g146067			1.10	−0.42	0.35	
Cre01.g057050.t1.1	Cre03.g144324			1.10	0.04	0.48	
Cre06.g304950.t1.1	Cre06.g304950			1.07	−0.65	0.30	
Cre08.g358200.t1.2	Cre08.g358200	*A4*	Protein kinase	1.07	−0.82	0.27	
Cre16.g689550.t1.2	Cre16.g689550	*PTK8*	Putative tyrosine kinase	1.06	−0.17	0.43	
Cre17.g720950.t1.1	Cre17.g720950		3-oxo-5-alpha-steroid 4-dehydrogenase	1.05	−0.26	0.40	
Cre02.g090950.t1.2	Cre02.g090950			1.05	−0.27	0.40	
Cre16.g683350.t1.1	Cre16.g683350			1.03	−0.67	0.31	
Cre02.g109450.t1.1	Cre02.g109450			1.01	−0.03	0.48	
Cre16.g652750.t1.1	Cre16.g652750			1.01	−0.29	0.41	
Cre03.g190000.t1.1	Cre03.g190000			1.00	−0.99	0.25	

* Data were ordered by FC in WT.

† Of the 52 most highly induced genes in WT (the top 10%), 37 were SAK1-dependent, and the induction of 33 of these genes was strongly attenuated to only 0.01-0.25 of magnitude of FC found in the WT. Dashed line indicates cutoff of FC for the top 10% most strongly induced genes.

‡ Genes that are repressed at basal level in *sak1.*

NF, not found in v5.

Classes of up-regulated genes in *sak1* were distinct from those of WT and included secondary metabolism of isoprenoids (Figure 3C; Table 6), precursors to photoprotective pigments such as carotenoids and tocopherols (Li et al., 2009). Phenylpropanoids, a group of metabolites associated with defense against stresses such as ultraviolet light and herbivores (Maeda and Dudareva, 2012), also represented a larger part of the response in *sak1* as compared to WT (Figure 3C). Another mutant-specific class of genes was cell vesicular transport, suggesting alteration in cell organization in response to the loss of *SAK1* (Figure 3C; Table 6). There were 434 genes that were down-regulated by ^1^O_2_ in the *sak1* mutant (Supplementary file 1, C2), none of which overlapped with the set of down-regulated genes in WT, in contrast to the overlap of up-regulated genes in the two genotypes (Figure 3A). Enriched classes of genes included those involved in DNA, nucleotide metabolism, hormone metabolism (not of brassinosteroid) and tetrapyrrole metabolism (Figure 3C, Table 6).

**Table 6.** Enriched functional classes among differentially expressed genes in *sak1* during ^1^O_2_ acclimation **DOI:** http://dx.doi.org/10.7554/eLife.02286.012

Primary Mapman class	Secondary Mapman class	Gene ID (v4)	Gene name	Annotation
Up-regulated genes				
Secondary metabolism	isoprenoids	Cre13.g565650.t1.1		Geranylgeranyl pyrophosphate synthase/Polyprenyl synthetase
		Cre06.g267600.t1.1		Lycopene epsilon cyclase
		Cre09.g407200.t1.1		Phytoene desaturase
		Cre06.g267600.t1.1		Lycopene epsilon cyclase
		Cre01.g011100.t1.1		Prenyltransferase and squalene oxidase repeat, Oxidosqualene-lanosterol cyclase and related proteins
	N misc	Cre08.g381707.t1.1		NF*
	phenylpropanoids	Cre03.g207800.t1.1		Alcohol dehydrogenase, class V
		Cre14.g623650.t1.1		Alcohol dehydrogenase, class V (Zinc-binding)
		Cre01.g039350.t1.1		Cytochrome P450 reductase, possibly CYP505B family
	sulfur-containing	Cre06.g299400.t1.1		NF*
	wax	Cre17.g722150.t1.1	*PKS3*	Type III polyketide synthase
		Cre07.g318500.t1.2		FAE1/Type III polyketide synthase-like protein, Chalcone and stilbene synthases
Lipid metabolism	‘exotics’ (steroids, squalene etc)	Cre01.g061750.t1.1		serine palmitoyltransferase
		Cre02.g137850.t1.1		NF*
		Cre83.g796250.t1.1		NF*
		Cre01.g011100.t1.1		Prenyltransferase and squalene oxidase repeat, Oxidosqualene-lanosterol cyclase and related proteins
	FA synthesis and FA elongation	Cre06.g256750.t1.1		Acyl carrier protein thioesterase
		Cre03.g182050.t1.1		Long-chain acyl-CoA synthetases (AMP-forming)
		Cre02.g074650.t1.1		Kelch repeat-containing proteins, Acyl-CoA binding protei
	glycerol metabolism	Cre01.g053000.t1.1	*GPD2*	Glycerol-3-phosphate dehydrogenase/dihydroxyacetone-3-phosphate reductase
	glycolipid synthesis	Cre13.g583600.t1.1	*DGD1*	Digalactosyldiacylglycerol synthase
	lipid degradation	Cre01.g057450.t1.2		NF*
		Cre02.g126050.t1.1		NF*
	phospholipid synthesis	Cre06.g281250.t1.1	*CFA1*	Cyclopropane fatty acid synthase
		Cre01.g038250.t1.1	*SDC1*	Serine decarboxylase
		Cre11.g472700.t1.1		NF*
		Cre13.g604700.t1.2		CDP-alcohol phosphatidyltransferase/Phosphatidylglycerol-phosphate synthase
Cell	vesicle transport	Cre18.g744100.t1.1		NF*
		Cre17.g721900.t1.1	*COG5*	Component of oligomeric golgi complex
		Cre01.g003050.t1.1	*SEC8*	Component of the Exocyst Complex
		Cre04.g224800.t1.1		Endosomal R-SNARE protein, Vamp7/Nyv1-family
		Cre17.g728150.t1.1		Endosomal R-SNARE protein, Yky6-family
		Cre12.g507450.t1.1		Trans-Golgi network Qa-SNARE protein, Syntaxin16/Syx16/Tlg2/Syp4-family
		Cre03.g210600.t1.1		NF*
		Cre04.g225900.t1.1		Endosomal R-SNARE protein, Vamp7/Nyv1-family
		Cre02.g101400.t1.1	*CHC1*	Clathrin Heavy Chain
		Cre17.g709350.t1.1		Late endosomal Qc-SNARE protein, Syx8/Syntaxin8-family
		Cre07.g342050.t1.1		Endosomal Qb-SNARE, Npsn-family
		Cre16.g692050.t1.1		ER-Golgi Qa-SNARE protein, Syntaxin5/Syx5/Sed5/Syp3-family
		Cre16.g676650.t1.1	*AP1G1*	Gamma1-Adaptin
		Cre02.g099000.t1.1		Late endosomal Qc-SNARE protein, Syx6/Tlg1/Syp5/6-family
		Cre12.g554200.t1.2		ER-Golgi Qb-SNARE, Memb/GS35/Bos1-family
		Cre06.g310000.t1.1	*AP4E1*	Epsilon4-Adaptin
		Cre10.g421250.t1.1	*EXO70*	Hypothetical Conserved Protein. Similar to Exo70, a subunit of the exocyst complex
		Cre07.g330950.t1.1	*AP4S4*	Sigma4-Adaptin
		Cre12.g488850.t1.2		Adaptin, alpha/gamma/epsilon
	division	Cre06.g269950.t1.1	*CDC48*	Protein involved in ubiquitin-dependent degradation of ER-bound substrates
		Cre08.g359200.t1.2		Regulator of chromosome condensation (RCC1)
	organisation	Cre13.g588600.t1.2		Kinesin (SMY1 subfamily)
		Cre12.g513450.t1.1	*TUH1*	Eta-Tubulin
		Cre01.g010950.t1.2		26S proteasome regulatory complex, subunit PSMD10 (Ankyrin repeat)
		Cre16.g679650.t1.2		Fimbrin/Plastin
		Cre06.g261950.t1.1		Myotrophin and similar proteins (Ankyrin repeat)
		Cre06.g291700.t1.1	*RSP3*	Radial spoke protein 3
		Cre10.g446700.t1.1	*ANK28*	Ankyrin repeat and DHHC-type Zn-finger domain containing proteins
Hormone metabolism†	abscisic acid	Cre16.g657800.t1.2	*CCD3*	Carotenoid cleavage dioxygenase
	auxin	Cre14.g609900.t1.1		Predicted membrane protein, contains DoH and Cytochrome b-561/ferric reductase transmembrane domains
	brassinosteroid	Cre16.g663950.t1.1		Sterol C5 desaturase
		Cre02.g092350.t1.1		Cytochrome P450, CYP51 superfamily; sterol 14 desaturase
		Cre12.g557900.t1.1	*CDI1*	C-8,7 sterol isomerase
		Cre02.g076800.t1.1		Delta14-sterol reductase, mitochondrial
		Cre12.g500500.t1.2		24-methylenesterol C-methyltransferase
	ethylene	Cre02.g108450.t1.1	*FAP280*	Flagellar Associated Protein, transcriptional coactivator-like, putative transcription factor
	jasmonate	Cre19.g756100.t1.1		NF*
Misc	acid and other phosphatases	Cre09.g396900.t1.1		NADH pyrophosphatase I of the Nudix family of hydrolases
		Cre06.g259650.t1.1		Calcineurin-like phosphoesterase, Acid-phosphatase-related
		Cre06.g249800.t1.1		Sphingomyelin synthetase -related
	cytochrome P450	Cre05.g234100.t1.1		Cytochrome P450, CYP197 superfamily
	dynamin	Cre02.g079550.t1.1	*DRP2*	Dynamin-related GTPase, involved in circadian rhythms
		Cre05.g245950.t1.1	*DRP1*	Dynamin-related GTPase
	glutathione S transferases	Cre03.g154950.t1.1		Glutathione S-transferase
	misc2	Cre12.g538450.t1.1	*EPT1*	CDP-Etn:DAG Ethanolamine phosphotransferase
	short chain dehydrogenase/reductase (SDR)	Cre12.g556750.t1.2		Short-chain dehydrogenase/reductase
		Cre08.g384864.t1.1		SH3 domain, protein binding
		Cre27.g775000.t1.1		NF*
		Cre17.g731350.t1.2		Short chain dehydrogenase
	UDP glucosyl and glucoronyl transferases	Cre02.g111150.t1.2	*ELG26*	Exostosin-like glycosyltransferase
		Cre02.g144050.t1.1		Acetylglucosaminyltransferase EXT1/exostosin 1
		Cre03.g204050.t1.2	*ELG6*	Exostosin-like glycosyltransferases
		Cre11.g474450.t1.1		NF*
		Cre03.g173300.t1.1		Lactosylceramide 4-alpha-galactosyltransferase (alpha- 1,4-galactosyltransferase)
		Cre02.g116600.t1.1	*ELG23*	Exostosin-like glycosyltransferase
Down-regulated genes				
Hormone metabolism†	cytokinin	Cre18.g744950.t1.2		NF*
		Cre16.g678900.t1.1		Response regulator receiver domain
		Cre01.g040450.t1.1	*HDT1*	Histidine-aspartic acid phosphotransferase 1 (phosphorylation cascade)
	ethylene	Cre09.g403550.t1.1		Iron/ascorbate family oxidoreductases
Nucleotide metabolism	deoxynucleotide metabolism	Cre12.g491050.t1.1	*RIR2*	Ribonucleotide reductase (RNR), small subunit
		Cre12.g492950.t1.1	*RIR1*	Ribonucleotide reductase (RNR), large subunit, class I
		Cre16.g667850.t1.1		dUTP pyrophosphatase
	synthesis	Cre14.g614300.t1.1		Inosine-5-monophosphate dehydrogenase/GMP reductase
		Cre07.g318750.t1.1		Phosphoribosylformylglycinamidine cyclo-ligase
Tetrapyrrole synthesis	porphobilinogen deaminase	Cre16.g663900.t1.1		Porphobilinogen deaminase
	protochlorophyllide reductase	Cre01.g015350.t1.1		Light-dependent protochlorophyllide reductase
	urogen III methylase	Cre02.g133050.t1.2		NF*
DNA	repair	Cre16.g670550.t1.2		XP-G/RAD2 DNA repair endonuclease
	synthesis/chromatin structure	Cre07.g338000.t1.1	*MCM2*	Minichromosome maintenance protein
		Cre07.g314900.t1.2		ATP-dependent RNA helicase, DEAD/DEAH helicase
		Cre03.g172950.t1.1	*CBF5*	Centromere/microtubule binding protein
		Cre01.g015250.t1.1		Eukaryotic DNA polymerase delta
		Cre27.g774200.t1.2		NF*
		Cre07.g316850.t1.1	*MCM4*	Minichromosome maintenance protein
	unspecified	Cre10.g451250.t1.2		Adenylate and guanylate cyclase catalytic domain, 3-5 exonuclease
		Cre01.g059950.t1.2		NF*

* Corresponding gene model was not found in v5.

† Functional terms are inferred by homology to the annotation set of *Arabidopsis thaliana* (Lopez et al., 2011).

To better understand the physiology of *sak1,* including the primary and secondary effects of lacking SAK1, we also focused on changes in transcript levels at the basal level, that is, without ^1^O_2_ treatment. At basal level 699 genes were induced, and 737 genes were repressed in the mutant compared to WT (Supplementary file 1, C3), displaying the genome-wide response to the loss of *SAK1* function despite the mutant’s wild-type appearance under normal lab growth conditions (Figure 1D). The enriched classes of genes that are differentially expressed are shown in Figure 3D. Genes induced in the mutant at basal level were enriched for those annotated to be involved in nucleotide metabolism, DNA, and RNA (Figure 3D; Table 7). Interestingly genes involved in tetrapyrrole and photosynthesis were enriched both in elevated and repressed genes at the basal level in *sak1*. There was no overall trend of these two pathways being up- or down-regulated, since these genes were at different steps of the pathway or encoded a select isoform of an enzyme or a subunit of a complex (Figure 3D; Table 7).

**Table 7.** Enriched functional classes among differentially expressed genes in *sak1* at basal level **DOI:** http://dx.doi.org/10.7554/eLife.02286.013

Primary Mapman class	Secondary Mapman class	Gene ID (v4)	Gene name	Annotation
Elevated in *sak1*				
nucleotide metabolism	deoxynucleotide metabolism	Cre12.g491050.t1.1	*RIR2*	Ribonucleotide reductase (RNR), small subunit
		Cre12.g492950.t1.1	*RIR1*	Ribonucleotide reductase (RNR), large subunit, class I
		Cre16.g667850.t1.1		dUTP pyrophosphatase
	phosphotransfer and pyrophosphatases	Cre02.g122450.t1.1		NF*
		Cre02.g093950.t1.1	*PYR5*	Uridine 5'- monophosphate synthase/orotate phosphoribosyltransferase
		Cre12.g519950.t1.1		Flagellar Associated Protein similar to adenylate/guanylate kinases
		Cre26.g772450.t1.1		NF*
	synthesis	Cre65.g793400.t1.1		NF*
		Cre02.g079700.t1.1	*PYR2*	Aspartate carbamoyltransferase
		Cre01.g048950.t1.1		dUTP pyrophosphatase
		Cre07.g318750.t1.1		Phosphoribosylformylglycinamidine cyclo-ligase.
DNA	repair	Cre07.g314650.t1.1		Chloroplast RecA recombination protein
	synthesis/chromatin structure	Cre04.g214350.t1.2		Eukaryotic DNA polymerase alpha, catalytic subunit
		Cre07.g314900.t1.2		ATP-dependent RNA helicase (DEAD/DEAH)
		Cre04.g223850.t1.1		Cytoplasmic DExD/H-box RNA helicase
		Cre01.g015250.t1.1		Eukaryotic DNA polymerase delta, catalytic subunit.
		Cre07.g342506.t1.1		Ubiquitin-protein ligase
		Cre07.g338000.t1.1	*MCM2*	Minichromosome maintenance protein
		Cre03.g178650.t1.1	*MCM6*	MCM6 DNA replication protein
		Cre07.g312350.t1.2		DNA polymerase alpha, primase subunit
		Cre01.g009250.t1.2	*TOP2*	DNA topoisomerase II
		Cre26.g772150.t1.1		NF*
		Cre07.g316850.t1.1	*MCM4*	Minichromosome maintenance protein 4
		Cre06.g263800.t1.2		tRNA-splicing endonuclease positive effector (SEN1)
		Cre06.g295700.t1.2	*MCM3*	Minichromosome maintenance protein
		Cre06.g251800.t1.1	*RFC4*	DNA replication factor C complex subunit 4
	unspecified	Cre07.g322300.t1.2		DNA repair helicase of the DEAD superfamily
		Cre17.g718100.t1.1		Phosphatidylinositol transfer protein SEC14 and related proteins (CRAL/TRIO)
Tetrapyrrole synthesis	Glu-tRNA reductase	Cre07.g342150.t1.1	*HEM1*	Glutamyl-tRNA reductase
	Glu-tRNA synthetase	Cre44.g788000.t1.1		Glutamyl-tRNA reductase
		Cre06.g306300.t1.1	*CHLI1*	Magnesium chelatase subunit I
	magnesium chelatase	Cre07.g325500.t1.1		Magnesium chelatase subunit H
	protochlorophyllide reductase	Cre01.g015350.t1.1	*POR1*	Light-dependent protochlorophyllide reductase
Photosynthesis	Calvin-Benson cycle	Cre05.g234550.t1.1		Fructose-biphosphate aldolase
	light reaction	Cre07.g330250.t1.1	*PSAH*	Subunit H of photosystem I
		Cre07.g334550.t1.1		Photosystem I subunit PsaO
		Cre06.g261000.t1.1	*PSBR*	10 kDa photosystem II polypeptide
	photorespiration	Cre12.g542300.t1.1	*GYK1*	Glycerate kinase
		Cre06.g253350.t1.1	*GCSH*	Glycine cleavage system, H-protein
		Cre06.g293950.t1.1	*SHMT2*	Serine hydroxymethyltransferase 2
Transport	ABC transporters and multidrug resistance systems	Cre04.g222700.t1.1		ATPase component of ABC transporters with duplicated ATPase domains/Translation elongation factor EF-3b
		Cre17.g728400.t1.2		ABCtransporter (ABC-2 type)
		Cre05.g241350.t1.2		ABCtransporter (ABC-2 type)
		Cre03.g169300.t1.1		ABCtransporter (ABC-2 type)
		Cre11.g474600.t1.1		NF*
	amino acids	Cre04.g226150.t1.2	*AOC1*	Amino acid carrier 1; belongs to APC (Amino acid Polyamine organo Cation) family
	calcium	Cre09.g388850.t1.1	*ACA1*	P-type ATPase/cation transporter, plasma membrane
	metabolite transporters at the envelope membrane	Cre06.g263850.t1.2	*TPT2*	Triose phosphate/phosphate translocator
	metabolite transporters at the mitochondrial membrane	Cre10.g449100.t1.1		Mitochondrial oxodicarboxylate carrier protein
		Cre01.g069350.t1.1		NF*
		Cre15.g641200.t1.1		Mitochondrial fatty acid anion carrier protein/Uncoupling protein
		Cre09.g396350.t1.1		Mitochondrial carrier protein PET8
	misc	Cre06.g311000.t1.2	*FBT2*	Folate transporte
		Cre17.g718100.t1.1		Phosphatidylinositol transfer protein SEC14 and related proteins (CRAL/TRIO)
	phosphate	Cre16.g686750.t1.1	*PTA3*	Proton/phosphate symporter
		Cre16.g675300.t1.2		Sodium-dependent phosphate transporter, major facilitator superfamily
	potassium	Cre12.g553450.t1.2		NF*
	sulphate	Cre17.g723350.t1.1	*SUL2*	Sulfate anion transporter
	unspecified cations	Cre13.g573900.t1.1		Na+:iodide/myo-inositol/multivitamin symporters
	sugars	Cre16.g675300.t1.2		Sodium-dependent phosphate transporter, major facilitator superfamily
RNA	processing	Cre10.g427700.t1.1		ATP-dependent RNA helicase, DEAD/DEAH box helicase
		Cre12.g538750.t1.1	*LSM1*	U6 snRNA-associated Sm-like protein LSm1, RNA cap binding; (SMP6d)
		Cre10.g433750.t1.2	*PAP1*	Nuclear poly(A) polymerase
		Cre03.g182950.t1.1		NF*
		Cre08.g375128.t1.1		NF*
	regulation of transcription	Cre17.g728200.t1.2		YL-1 protein (transcription factor-like 1)
		Cre06.g275500.t1.1		AP2 Transcription factor
		Cre28.g777500.t1.2		NF*
		Cre13.g572450.t1.1		Response regulator receiver domain (sensor histidine kinase-related, regulation of transcription)
		Cre14.g620500.t1.1		AP2 Transcription factor
		Cre16.g673150.t1.1		Histone deacetylase complex, catalytic component RPD3
		Cre02.g078700.t1.2		DNA damage-responsive repressor GIS1/RPH1, jumonji superfamily
		Cre03.g198800.t1.1		Myb-like DNA-binding domain
		Cre04.g218050.t1.2		RWP-RK domain
		Cre07.g324400.t1.1	*VPS24*	Subunit of the ESCRT-III complex, vaculoar sortin protein
		Cre11.g481050.t1.1		SWI/SNF-related chromatin binding protein
		Cre02.g101950.t1.1	*TMU2*	tRNA (uracil-5)-methyltransferase
		Cre10.g459600.t1.2		CAATT-binding transcription factor/60S ribosomal subunit biogenesis protein
		Cre01.g018650.t1.2		NF*
		Cre01.g012200.t1.2		NF*
		Cre02.g129750.t1.1		NF*
		Cre10.g461750.t1.2		DNA (cytosine-5-)-methyltransferase
		Cre01.g004600.t1.2	*RWP12*	Putative RWP-RK domain transcription factor
		Cre09.g400100.t1.1		Predicted Zn-finger protein, zinc and DNA binding domains
		Cre07.g335150.t1.2		SBP domain
	RNA binding	Cre16.g662700.t1.1		NF*
		Cre07.g330300.t1.1		RNA-binding protein musashi/mRNA cleavage and polyadenylation factor I complex, subunit HRP1
		Cre06.g275100.t1.1		RNA-binding protein musashi/mRNA cleavage and polyadenylation factor I complex, subunit HRP1
	transcription	Cre07.g322200.t1.1		NF*
				
Repressed in *sak1*				
Transport	ABC transporters and multidrug resistance systems	Cre02.g097800.t1.2		ABC transporter (MDR)
		Cre17.g725200.t1.1		ABC transporter, peptide exporter
		Cre13.g580300.t1.1		ABC transporter family protein
		Cre10.g439000.t1.2		Long-chain acyl-CoA transporter, ABC superfamily (involved in peroxisome organization and biogenesis)
	amino acids	Cre06.g292350.t1.1	*AOC4*	Amino acid carrier
	calcium	Cre06.g263950.t1.2		Sodium/potassium-transporting ATPase subunit alpha
		Cre16.g681750.t1.2		Calcium transporting ATPase
	metabolite transporters at the mitochondrial membrane	Cre03.g172300.t1.1		Mitochondrial phosphate carrier protein
		Cre09.g394800.t1.2		Mitochondrial substrate carrier protein
	metal	Cre03.g189550.t1.2	*ZIP3*	Zinc transporter, ZIP family
		Cre11.g479600.t1.2		Sodium/calcium exchanger NCX1 and related proteins
		Cre06.g281900.t1.1	*ZIP7*	Zinc transporter and related ZIP domain-containing proteins
	misc	Cre02.g089900.t1.1		Secretory carrier membrane protein
		Cre10.g448050.t1.1		Retinaldehyde binding protein-related (CRAL/TRIO domain)
		Cre03.g177750.t1.2		Multidrug resistance pump
	NDP-sugars at the ER	Cre02.g112900.t1.1		GDP-fucose transporter (Triose-phosphate transporter family)
	P- and V-ATPases	Cre01.g027800.t1.1	*ATPvH*	Vacuolar ATP synthase subunit H
		Cre10.g446550.t1.1	*ATPvF*	Vacuolar ATP synthase subunit F
		Cre03.g176250.t1.1	*ATPvD1*	Vacuolar ATP synthase subunit D
		Cre06.g250250.t1.1	*ATPvC*	Vacuolar ATP synthase subunit C
		Cre10.g459200.t1.1	*ACA4*	P-type ATPase/cation transporter, plasma membrane (Low CO2 inducible gene)
	phosphate	Cre12.g515750.t1.2		Sodium-dependent phosphate transporter-related
		Cre08.g379550.t1.2		Sodium-dependent phosphate transporter, major facilitator superfamily
		Cre12.g489400.t1.1	*PTB7*	Putative phosphate transporter, sodium/phosphate transporter
		Cre02.g144650.t1.1	*PTB12*	Sodium/phosphate symporter
	unspecified anions	Cre09.g404100.t1.1		Cl- channel CLC-7 and related proteins (CLC superfamily)
		Cre17.g729450.t1.1		Cl- channel CLC-7 and related proteins (CLC superfamily)
		Cre01.g037150.t1.2		Voltage-gated chloride channel activity
	sugars	Cre03.g206800.t1.2	*HXT1*	Hexose transporter
	P- and V-ATPases	Cre03.g176250.t1.1	*ATPvD1*	Vacuolar ATP synthase subunit D
		Cre10.g446550.t1.1	*ATPvF*	Vacuolar ATP synthase subunit F
		Cre01.g027800.t1.1	*ATPvH*	Vacuolar ATP synthase subunit H
Mitochondrial electron transport / ATP synthesis	cytochrome c reductase	Cre01.g051900.t1.1	*RIP1*	Rieske iron-sulfur protein of mitochondrial ubiquinol-cytochrome c reductase (complex III)
		Cre06.g262700.t1.2		Ubiquinol cytochrome c reductase, subunit 7
	F_1_-ATPase	Cre02.g116750.t1.2		F0F1-type ATP synthase, alpha subunit
		Cre01.g018800.t1.1	*ATP6*	Mitochondrial F1F0 ATP synthase subunit 6
		Cre10.g420700.t1.1		Mitochondrial F1F0-ATP synthase, subunit epsilon/ATP15
		Cre16.g680000.t1.1	*ATP5*	Mitochondrial ATP synthase subunit 5, OSCP subunit
	NADH-DH	Cre10.g434450.t1.1	*NUOA9*	Putative NADH:ubiquinone oxidoreductase (Complex I) 39 kDa subunit
		Cre08.g378900.t1.1	*NUO3*	NADH:ubiquinone oxidoreductase ND3 subunit
		Cre10.g450400.t1.1	*NUO5*	NADH:ubiquinone oxidoreductase (Complex I) 24 kD subunit
Lipid metabolism	'exotics' (steroids, squalene etc)	Cre14.g615050.t1.1		3-oxo-5-alpha-steroid 4-dehydrogenase, Steroid reductase required for elongation of the VLCFAs (enoyl reductase)
		Cre12.g530550.t1.2	*KDG2*	Diacylglycerol kinase, sphingosine kinase
		Cre02.g137850.t1.1		NF*
	FA desaturation	Cre17.g711150.t1.1		Omega-6 fatty acid desaturase (delta-12 desaturase)
	glyceral metabolism	Cre13.g577450.t1.2		Glycerol-3-phosphate dehydrogenase
	glycolipid synthesis	Cre13.g583600.t1.1	*DGD1*	Digalactosyldiacylglycerol synthase
		Cre16.g656400.t1.1	*SQD1*	UDP-sulfoquinovose synthase
	lipid degradation	Cre06.g252801.t1.2		CGI-141-related/lipase containing protein (TAG lipase)
		Cre03.g164350.t1.2		Lysophospholipase, putative drug exporter of the RND superfamily
	phospholipid synthesis	Cre06.g281250.t1.1	*CFA1*	Cyclopropane fatty acid synthase
		Cre09.g398700.t1.1	*CFA2*	Cyclopropane fatty acid synthase
		Cre11.g472700.t1.1		NF*
		Cre06.g262550.t1.1		Zinc finger MYND domain containing protein 10
Photosynthesis	Calvin-Benson cycle	Cre12.g511900.t1.1	*RPE1*	Ribulose phosphate-3-epimerase
		Cre02.g120100.t1.1	*RBCS1*	Ribulose-1,5-bisphosphate carboxylase/oxygenase small subunit 1
	light reaction	Cre05.g243800.t1.1	*CPLD45*	Photosystem II Psb27 protein
		Cre10.g420350.t1.1	*PSAE*	Photosystem I reaction center subunit IV
		Cre01.g071450.t1.2		NF*
		Cre06.g291650.t1.1		Ferredoxin
		Cre05.g242400.t1.1		No functional annotation
	photorespiration	Cre09.g411900.t1.2	*SHMT3*	Serine hydroxymethyltransferase 3
		Cre06.g295450.t1.1	*HPR1*	Hydroxypyruvate reductase
Major CHO metabolism	degradation	Cre09.g415600.t1.2		Starch binding domain
		Cre11.g473500.t1.2		NF*
		Cre09.g415600.t1.2		Starch binding domain
	synthesis	Cre06.g289850.t1.2	*SBE1*	Starch Branching Enzyme
		Cre17.g721500.t1.1		Granule-bound starch synthase I
misc	acid and other phosphatases	Cre13.g568600.t1.2		Multiple inositol polyphosphate phosphatase-related, Acid phosphatase activity
	alcohol dehydrogenases	Cre13.g569350.t1.1		Sterol dehydrogenase-related, Flavonol reductase/cinnamoyl-CoA reductase
	cytochrome P450	Cre07.g356250.t1.2		Cytochrome P450 CYP4/CYP19/CYP26 subfamilies, beta-carotene 15,15'-monooxygenase
		Cre07.g356250.t1.2		Cytochrome P450 CYP4/CYP19/CYP26 subfamilies, beta-carotene 15,15'-monooxygenase
	dynamin	Cre17.g724150.t1.1	*DRP3*	Dynamin-related GTPase
	GCN5-related N-acetyltransferase	Cre16.g657150.t1.2		N-acetyltransferase activity (GNAT) family
	gluco-, galacto- and mannosidases	Cre03.g171050.t1.2	*GHL1*	Glycosyl hydrolase
	misc2	Cre14.g614100.t1.1	*GTR26*	Dolichyl-diphosphooligosaccharide-protein glycosyltransferase
	rhodanese	Cre07.g352550.t1.1	*RDP3*	Putative rhodanese domain phosphatase
	short chain dehydrogenase/reductase (SDR)	Cre07.g352450.t1.1		Corticosteroid 11-beta-dehydrogenase and related short chain-type dehydrogenases, 3-hydroxybutyrate dehydrogenase
		Cre12.g559350.t1.1		1-Acyl dihydroxyacetone phosphate reductase and related dehydrogenases
		Cre03.g191850.t1.1		Short chain dehydrogenase
	UDP glucosyl and glucoronyl transferases	Cre11.g474450.t1.1		NF*
		Cre03.g205250.t1.2	*ELG4*	Exostosin-like glycosyltransferase
		Cre16.g659500.t1.1		Lactosylceramide 4-alpha-galactosyltransferase
		Cre11.g483400.t1.2	*ELG10*	Exostosin-like glycosyltransferase
Tetrapyrrole synthesis	Glu-tRNA synthetase	Cre12.g510800.t1.1	*CHLI2*	Magnesium-chelatase subunit chlI
	magnesium protoporphyrin IX methyltransferase	Cre12.g498550.t1.2		Magnesium protoporphyrin IX S-adenosyl methionine O-methyl transferase (Magnesium-protoporphyrin IX methyltransferase) (PPMT)
	unspecified	Cre12.g516350.t1.1	*COX10*	Cytochrome c oxidase assembly protein Cox10
	urogen III methylase	Cre02.g133050.t1.2		NF*

* Corresponding gene model was not found in v5.

**Figure 4.**
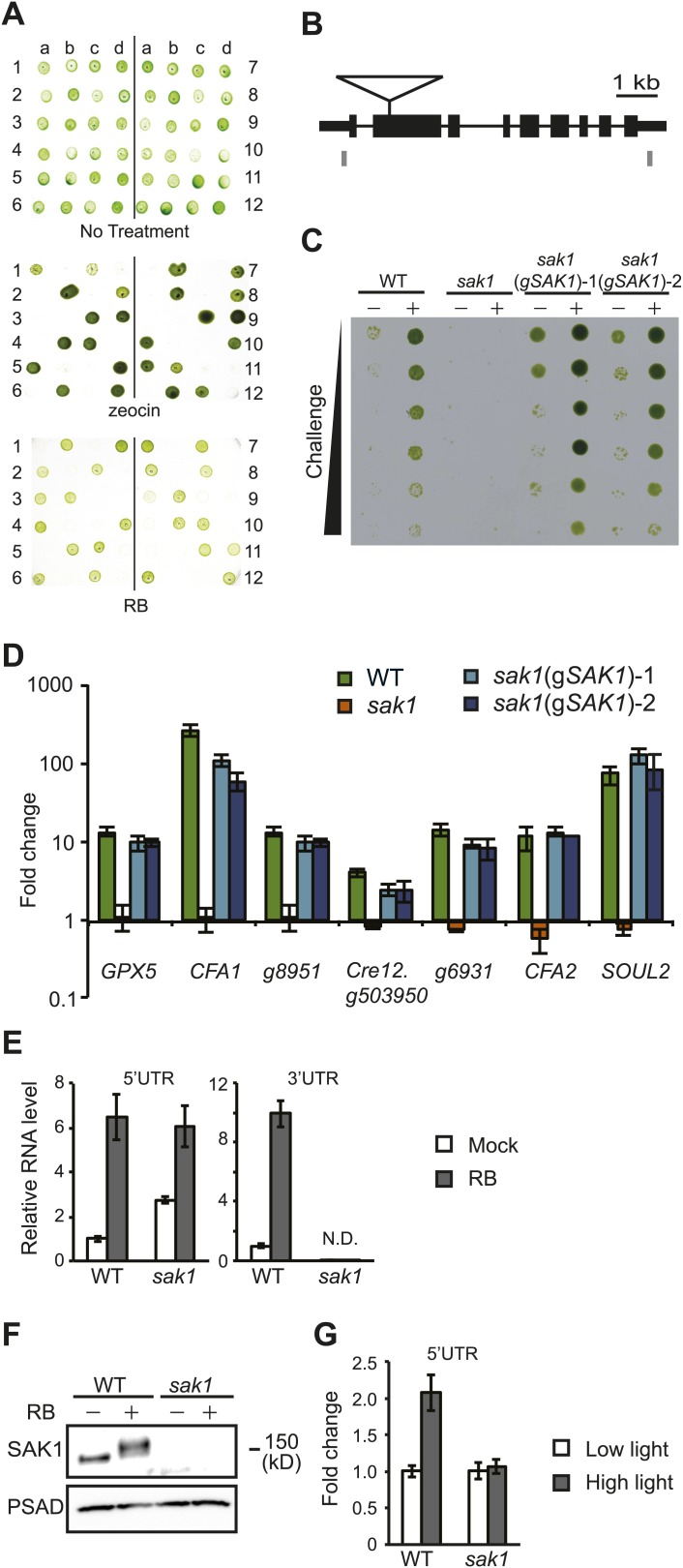
Genetic and molecular analysis of *sak1.* (**A**) The insertion of a zeocin resistance gene and the RB sensitivity phenotype are linked. Twelve complete tetrads from a backcross of *sak1* to wild type are shown. Numbers indicate independent tetrads, and letters (a-d) indicate the individual progeny from tetrads. (**B**) Gene structure of *SAK1* and the insertion site. Gray boxes indicate positions of primers used for qPCR. (**C**) Transformation of *sak1* with a genomic fragment containing *SAK1* rescues the acclimation phenotype. *sak1*(g*SAK1*)-1 and *sak1*(g*SAK1*)-2 are two independent transformants. (**D**) *sak1*(g*SAK1*)-1 and *sak1*(g*SAK1*)-2 show recovery of ^1^O_2_ target gene expression. Y-axis indicates fold change during acclimation to ^1^O_2_. (**E**) qRT-PCR of *SAK1* in WT and *sak1* mutant using primers for 5′- and 3′-UTR shown in panel **B**. (**F**) SAK1 protein is induced in WT and detected as higher molecular weight bands during acclimation to ^1^O_2_ generated by RB. (**G**) *SAK1* transcript probed for 5′-UTR in cells transferred from low light to high light for 1 hr. Error bars indicate standard deviation of biological triplicates. **DOI:** http://dx.doi.org/10.7554/eLife.02286.014

We observed that some of the genes more strongly dependent on *SAK1* had repressed transcript levels (e.g., *CFA1* and *SOUL2*), indicating that *SAK1* is required for their basal expression, while others had elevated basal levels (*GPX5*), suggesting that expression of these genes is controlled also by other pathways. As is discussed in the following section, *SAK1* expression monitored by qRT-PCR followed the latter trend as the 5′UTR of the gene was elevated in the mutant (Figure 4E), which may be a result of response to other factors such as a possible oxidization product of ^1^O_2_. The SAK1-dependent genes induced by ^1^O_2_ and repressed at basal level in the mutant (i.e., those that require *SAK1* for basal expression) are indicated in Table 5.

### The *sak1* mutant identifies a single nuclear gene that is itself induced during acclimation to ^1^O_2_

The *sak1* mutant was generated by insertional mutagenesis using a plasmid that confers resistance to zeocin (Dent et al., 2005). Progeny obtained from a backcross of *sak1* with WT showed that the mutation causing the RB sensitivity phenotype was linked to zeocin resistance (Figure 4A). The site of insertion was identified by thermal asymmetric interlaced (TAIL)-PCR (Liu et al., 1995) as the second exon of the annotated gene Cre17.g741300 on chromosome 17 (Figure 4B). To test whether this gene is responsible for the mutant phenotype, a genomic fragment containing the gene with an additional ∼500 bp region upstream of the predicted transcription start site was cloned and introduced into the mutant by co-transformation. Among the approximately 300 transformants screened, two clones appeared to have recovered the RB acclimation phenotype (Figure 4C). Furthermore, induction of genes we found attenuated in *sak1* (Figure 2) was restored in these transformants (Figure 4D), confirming that Cre17.g741300 is the *SAK1* gene required for acclimation and the gene expression response to ^1^O_2_.

In WT, the *SAK1* gene itself was induced by 6- to 10-fold during acclimation when probed for the 5′-and 3′-UTR of the transcript by qRT-PCR (Figure 4E). The mutant displayed elevated basal level and induction of the 5′-UTR during acclimation, whereas the 3′-UTR of the transcript was undetectable, indicating that the full-length transcript was absent in *sak1* (Figure 4E). An antibody raised against an epitope of the SAK1 protein detected a single band in basal conditions, whereas the SAK1 protein appeared as multiple bands with higher molecular weight in acclimated WT cells, all of which were absent in the mutant (Figure 4F). *SAK1* transcript was induced when probed for the 5′-UTR during high light exposure in both WT and *sak1* (Figure 4G) similarly to other ^1^O_2_-response genes identified by RNA-seq (Table 1), indicating that *SAK1* itself is part of the endogenous response to high light.

### SAK1 contains an uncharacterized domain conserved in chlorophytes and found in some bZIP transcription factors

**Figure 5.**
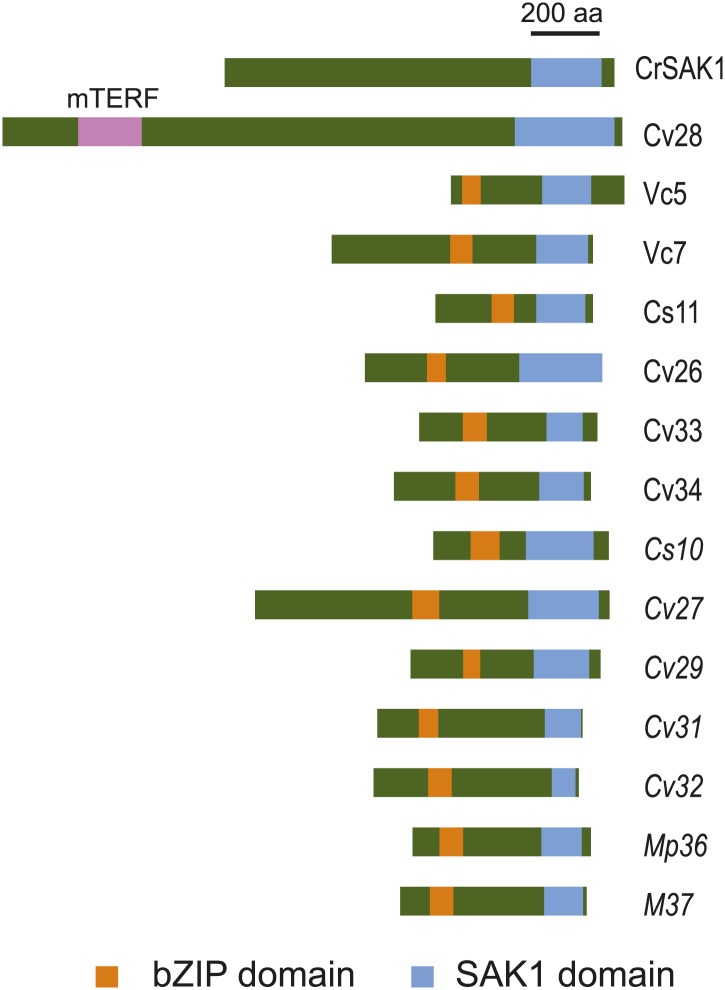
SAK1 contains an uncharacterized domain present in some bZIP transcription factors. Schematic of relative positions of SAK1 and bZIP domains. One protein (Cv28) contains a mitochondrial termination factor (mTERF) domain. The letters and numbers in the abbreviated names represent initials of the species and numbers listed in Table 8. Proteins with italicized names contain bZIP domains that were recognized by Pfam but scored below significance. **DOI:** http://dx.doi.org/10.7554/eLife.02286.016

The predicted SAK1 protein consists of 1141 amino acid residues and has no domains with functional annotation. Only a ∼150-residue region at the C-terminus, designated the SAK1 domain, has similarity to other proteins. Many predicted proteins within chlorophytes (*Volvox carteri* [8 proteins], *Coccomyxa subellipsoidea* [3 proteins], *Chlamydomonas* [14 proteins], *Chlorella variabilis* [9 proteins] and *Micromonas* [3 proteins]) (Table 8) contain this domain as shown in the alignment in Figure 5—figure supplement 1. Among the 37 members of the chlorophyte SAK1 domain family, 13 have possible bZIP transcription factor domains (six were significant Pfam hits and seven were below the threshold for significance but recognizable by Pfam) (Figure 5). One protein contained a mitochondrial (transcription) termination factor (mTERF) domain (Figure 5), defined by its three leucine zipper domains required for DNA binding (Fernandez-Silva et al., 1997). Proteins with more distantly related SAK1 domains were found by PSI-BLAST in plants, many of which were hypothetical or unknown proteins but also included bZIP transcription factors.

**Table 8.** SAK1 domain containing proteins in chlorophytes **DOI:** http://dx.doi.org/10.7554/eLife.02286.015

Number in alignment	Organism	Transcript/Protein IDaTranscript/Protein IDaTranscript/Protein ID*
1	*Volvox carteri*	Vocar20009235
2	*Volvox carteri*	Vocar20002437
3	*Volvox carteri*	Vocar20002672
4	*Volvox carteri*	Vocar20004923
5	*Volvox carteri*	Vocar20012349
6	*Volvox carteri*	Vocar20005988
7	*Volvox carteri*	Vocar20007158
8	*Volvox carteri*	Vocar20007883
9	*Coccomyxa subellipsoidea*	57405
10	*Coccomyxa subellipsoidea*	59655
11	*Coccomyxa subellipsoidea*	57694
12	*Chlamydomonas reinhardtii*	Cre16.g652650.t1.3
13	*Chlamydomonas reinhardtii*	Cre06.g271000.t1.2
14	*Chlamydomonas reinhardtii*	Cre06.g285800.t1.2
15	*Chlamydomonas reinhardtii*	Cre06.g275600.t1.2
16	*Chlamydomonas reinhardtii*	Cre06.g285750.t1.3
17	*Chlamydomonas reinhardtii*	Cre06.g270950.t1.2
18	*Chlamydomonas reinhardtii*	g9774.t1
SAK1	*Chlamydomonas reinhardtii*	KF985242
20	*Chlamydomonas reinhardtii*	Cre03.g179150.t1.2
21	*Chlamydomonas reinhardtii*	g3701.t1
22	*Chlamydomonas reinhardtii*	Cre03.g179250.t1.2
23	*Chlamydomonas reinhardtii*	Cre03.g179200.t1.2
24	*Chlamydomonas reinhardtii*	Cre01.g004800.t1.2
25	*Chlamydomonas reinhardtii*	Cre01.g048550.t1.3
26	*Chlorella variabilis*	EFN51260
27	*Chlorella variabilis*	EFN53496
28	*Chlorella variabilis*	EFN55618
29	*Chlorella variabilis*	EFN57652
30	*Chlorella variabilis*	EFN55658
31	*Chlorella variabilis*	EFN54262
32	*Chlorella variabilis*	EFN54510
33	*Chlorella variabilis*	EFN55806
34	*Chlorella variabilis*	EFN53492
35	*Micromonas sp. RCC299*	ACO61347
36	*Micromonas pusilla CCMP1545*	EEH57791
37	*Micromonas sp. RCC299*	ACO65814

* 1–25, as defined on phytozome.net; 26–37, CrSAK1, genbank accession numbers.

**Figure 5—figure supplement 1.**
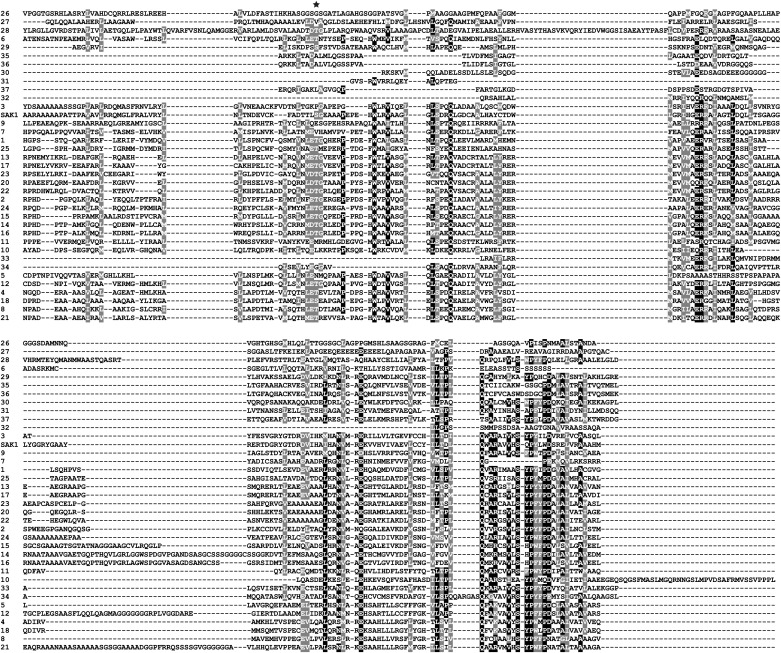
Multiple sequence alignment of SAK1 domains. The SAK1 domains of 37 chlorophyte proteins were aligned by MUSCLE (phylogeny.fr). Protein identities are as shown in Table 8. Star indicates a relatively conserved residue within the SAK1 domain that was predicted to be a possible phosphorylation site (Figure 5—figure supplement 3). **DOI:** http://dx.doi.org/10.7554/eLife.02286.017

**Figure 5—figure supplement 2.**
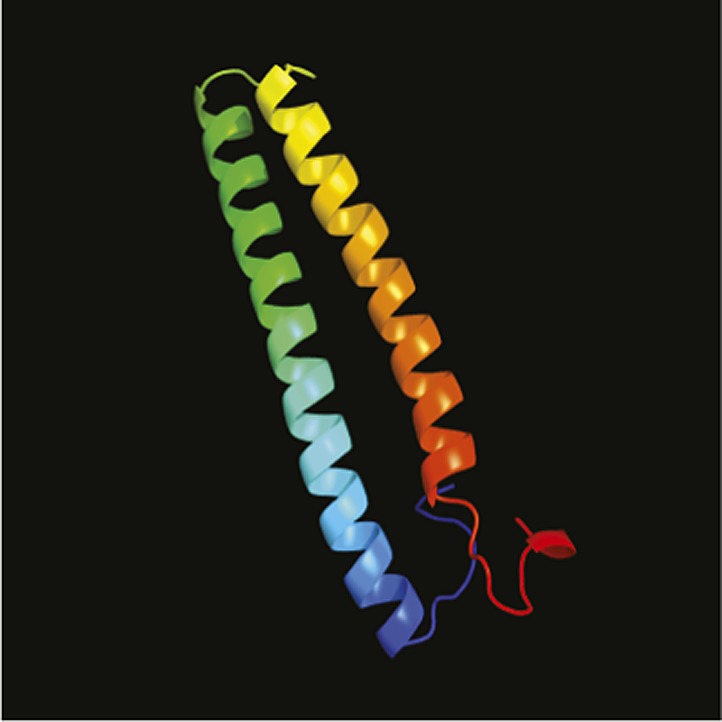
Secondary structure prediction of SAK1 domain. SAK1 domain modeled against its best-hit nickel cobalt resistance protein cnrr by PHYRE. 44% (coverage) of the SAK1 domain was aligned with 73.6% confidence. **DOI:** http://dx.doi.org/10.7554/eLife.02286.018

**Figure 5—figure supplement 3.**
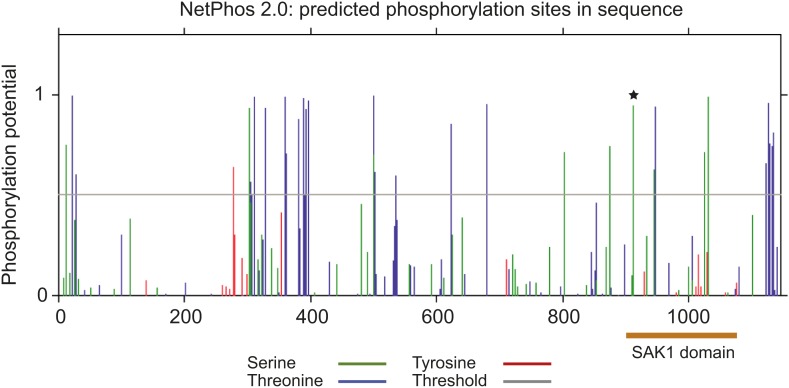
Prediction of phosphorylation sites in SAK1. Prediction of phosphorylation sites by NetPhos 2.0. Orange bar indicates the position of SAK1 domain, star indicates a relatively conserved residue among the 37 members containing the SAK1 domain. **DOI:** http://dx.doi.org/10.7554/eLife.02286.019

Amino acid positions 900 to 1089 of SAK1, corresponding to the region aligned with other proteins in Figure 5—figure supplement 1, were searched for secondary structure using PHYRE, and this region was predicted to consist of mostly alpha helices with some disordered intervals. The top hit was a cobalt/nickel-binding resistance protein cnrr, and 44% of the residues were modeled with 73.6% confidence (Figure 5—figure supplement 2).

### SAK1 resides mainly in the cytosol and is phosphorylated during induction by ^1^O_2_

**Figure 6.**
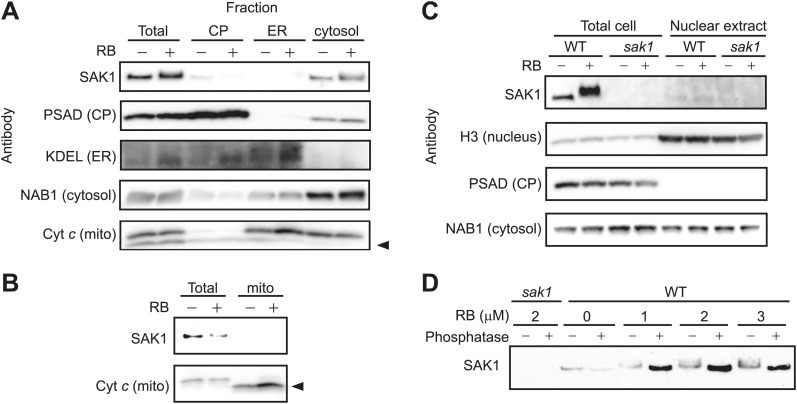
SAK1 is a phosphorylated protein that is in the cytosol. (**A** and **B**) SAK1 is detected in the cytosol and not in other subcellular fractions. (**C**) SAK1 is not enriched in nuclear extracts. Approximately 30 μg of protein was loaded into each well except for mitochondrial fractions that were loaded approximately 7.5 μg protein due to low protein yield in isolated fractions. Subcellular markers: Chloroplast (CP), PSAD; Endoplasmic reticulum (ER), KDEL; Cytosol, NAB1; Mitochondria (mito), cytochrome *c* (Cyt c); Nuclear, histone 3 (H3). The arrowhead indicates the band corresponding to Cyt *c*. (**D**) Protein extracts from cells treated with increasing concentrations of RB were then treated with phosphatase (+) or only with buffer (−) before detection of SAK1 by immunoblot analysis. **DOI:** http://dx.doi.org/10.7554/eLife.02286.020

To obtain insight into the function of SAK1, we isolated subcellular fractions enriched for chloroplast, ER, cytosol, and mitochondria from WT cells. The *Chlamydomonas* cell contains a single large chloroplast that is physically connected to other organelles such as the ER, making it particularly challenging to fractionate. The patterns of markers specific for chloroplast, ER, cytosol, and mitochondria showed that each target fraction was enriched as expected, although with some cross contamination (Figure 6A,B). The distribution of SAK1 in these fractions resembled most closely that of the cytosolic marker NAB1 (Mussgnug et al., 2005), although the SAK1 signal was not as enriched as NAB1 in the cytosolic fraction, possibly due to partial degradation of SAK1 during the fractionation. The localization was the same in cells with and without RB treatment (Figure 6A). Because *SAK1* was required for the induction of many genes during acclimation to ^1^O_2_ and the list of proteins with similarity to SAK1 included those predicted to be bZIP transcription factors, we tested whether SAK1 protein was dually targeted to the nucleus and cytosol, which would account for the lack of enrichment of SAK1 in the cytosolic fraction (Figure 6A). As shown in Figure 6C although a faint SAK1 signal was detected in nuclear fraction, there was no enrichment as seen for the nuclear marker histone H3 (H3). The distribution of the cytosolic marker NAB1 indicated the contamination of the nuclear fraction by cytosolic proteins (Figure 6C). Therefore we conclude that the low signal of SAK1 in the nuclear fraction is likely to be due to cytosolic contamination. Attempts to detect the protein by immunofluorescence using anti-SAK1 antibodies as well as anti-FLAG and anti-HA antibodies against tagged proteins in transgenic lines were unsuccessful due to a very low signal-to-noise ratio even in bleached cells.

By SDS-PAGE and immunoblot analysis, SAK1 appeared in multiple forms with higher molecular weight during acclimation compared to that observed in control cells (Figures 4F and 6A,C). When the extracted protein samples were treated with phosphatase, the diffuse pattern of multiple forms collapsed into a single band detected by immunoblot analysis that had an even higher mobility that that of untreated cells (Figure 6D). This result indicates that SAK1 is a phosphorylated protein during basal conditions, and it is further phosphorylated upon exposure of cells to ^1^O_2_.

## Discussion

### *SAK1* is necessary for acclimation of *Chlamydomonas* cells to ^1^O_2_

To understand the retrograde signal transduction pathway involved in the cellular response to ^1^O_2_, we focused on the unique ability of *Chlamydomonas* to acclimate to ^1^O_2_ stress (Ledford et al., 2007), and we isolated a regulatory mutant that is unable to acclimate. Several previous genetic screens aimed at dissecting the mechanisms of ^1^O_2_ signaling have concentrated on the nuclear gene expression response to ^1^O_2_, often relying on the response of a single marker gene (Baruah et al., 2009a; Brzezowski et al., 2012; Fischer et al., 2012; Shao et al., 2013). In contrast, our screen exploited a physiological response to sublethal levels of ^1^O_2_, which induces the wild type to survive a subsequent, otherwise lethal treatment with the ^1^O_2_ generator RB (Ledford et al., 2007). The *sak1* mutant completely lacks this ability to acclimate to ^1^O_2_ (Figure 1A). An analogous phenotype is exhibited by the *yap1Δ* mutant of *Saccharomyces cerevisiae*, which is unable to acclimate to hydrogen peroxide stress (Stephen et al., 1995).

In contrast to the complete loss of acclimation to RB, *sak1* acclimates (but less effectively than WT) when pretreated with high light and challenged with RB (Figure 1B). This result suggests that the high light pretreatment induces a broader response than that elicited by RB and that *sak1* is still able to respond to other signals besides ^1^O_2_ (e.g., plastoquinone redox state, H_2_O_2_, and/or superoxide) that are involved in the response to high light*.* When tested on TAP agar plates for photoheterotrophic growth in the presence of various photosynthetic inhibitors, the *sak1* mutant displayed sensitivity to RB but not to other inhibitors (Figure 1D). In particular, *sak1* is not more sensitive than WT to high light or norflurazon (an inhibitor of the biosynthesis of carotenoids, which function as quenchers of ^1^O_2_). We speculate that the lack of ^1^O_2_-sensitive phenotype in these plate experiments is attributable to the time-scale of the treatments involved. ^1^O_2_ generated by RB or during a transfer to higher light intensity is transient, whereas NF requires longer time to exert its effect because it needs to enter the cell, inhibit biosynthesis, and deplete cells of existing carotenoids. During this time, the cell is likely able to acclimate by detoxifying and reducing the generation of ^1^O_2_ by various means such as changing the composition of the photosynthetic apparatus. We have previously shown that acclimation to ^1^O_2_ is transient and is dissipated by 24 hr post-treatment (Ledford et al., 2007). Consistent with this, pretreatment with RB does not acclimate the cells to stresses such as growth in high light or norflurazon that require a period of days to assess an effect on viability (Figure 1—figure supplement 1). We have also observed that under our experimental conditions, the induction of target gene expression upon exposure to ^1^O_2_ lasts up to 90 min and then declines. We conclude that SAK1 functions mainly during transient perturbations that generate ^1^O_2_. However, during steady-state growth under high light or norflurazon, the cell is able to cope by other means that do not involve *SAK1.*

### *SAK1* is necessary for a subset of the genome-wide response to ^1^O_2_ in *Chlamydomonas*

A physiological acclimation response that results in such an evident growth phenotype (Figure 1A) likely involves large-scale changes in gene expression, and transcriptome analysis of wild-type cells showed that hundreds of nuclear genes are up- or down-regulated during acclimation to ^1^O_2_ (Figure 3A,B; Supplementary file 1, C1). The *sak1* mutant is specifically impaired in regulation of a notable subset of these genes, that is, those that are most strongly induced in the wild type (Table 5), suggesting that these genes play a key role in the acclimation response to ^1^O_2_.

In particular, many genes involved in sterol and lipid metabolism were induced by ^1^O_2_ in *Chlamydomonas* (Figure 3B; Table 3). For example, two genes encoding putative cyclopropane fatty acid synthase (*CFA1* and *CFA2*) exhibited SAK1-dependent induction (Figure 2). Cyclopropane fatty acids have been found in large amounts in the seeds of *Sterculia foetida* (Bao et al., 2002), although its biological function is unknown. In bacteria, it has been implicated in oxidative stress responses (Guerzoni et al., 2001; Kim et al., 2005) and particularly in the anoxygenic photosynthetic bacterium *Rhodobacter sphaeroides,* CFA gene expression is induced during ^1^O_2_ stress by a σ^E^ factor (Ziegelhoffer and Donohue, 2009). Interestingly CFA mutants of *R. sphaeroides* are compromised in the induction of genes in response to ^1^O_2_, suggesting a regulatory role of the gene, protein, or the product of its enzymatic function (cyclopropane fatty acids, Bao et al., 2002) in gene expression rather than solely a biochemical stress response (Nam et al., 2013).

Another intriguing class of up-regulated genes enriched during ^1^O_2_ acclimation in WT and not in *sak1* was a group of genes encoding transporters, especially ABC transporters related to the MDR and PDR types. This was not surprising considering that ^1^O_2_ exists in aquatic and terrestrial environments, where it is generated by photosensitizing humic substances (Frimmel et al., 1987; Steinberg et al., 2008), which are known to affect microbial populations including phytoplankton (Glaeser et al., 2010, 2014). Assuming that some of these transporters function to export photosensitizing molecules from the cell, our results suggest that removal of photosensitizers is an integral part of the ^1^O_2_ response in *Chlamydomonas*, rather than simply a response to the presence of a xenobiotic compound such as RB (Table 4). It is likely that *Chlamydomonas*, a soil-dwelling microalga, needs to respond to ^1^O_2_ that is generated not only in the chloroplast, but also in other compartments. In this context, it is noteworthy that a recent study has demonstrated light-independent ^1^O_2_ generation in multiple organelles other than the chloroplast under various biotic and abiotic stresses in plants (Mor et al., 2014).

Two proteins with SOUL heme-binding domains were among SAK1-dependent up-regulated genes (SOUL2 and Cre06.g299700.t1.1, formerly annotated as SOUL1 in v4). Aside from their ability to bind various porphyrins (Blackmon et al., 2002; Sato et al., 2004), SOUL heme-binding proteins have been described in diverse biological functions in mice, such as in apoptosis by interacting with a mitochondrial anti-apoptotic factor Bcl-xL (Ambrosi et al., 2011) or an isoform-specific role in retina and pineal gland (Zylka and Reppert, 1999). The latter form is suggested to play a role in transporting heme or by binding free heme to prevent oxidative stress (Sato et al., 2004). In *Arabidopsis* a chloroplast-localized SOUL5 protein has been shown to interact with a heme oxygenase, HY1, and mutation of the gene encoding SOUL5 causes oxidative stress (Lee et al., 2012). *Chlamydomonas* contains five putative SOUL heme-binding proteins, only one of which contains an amino-terminal chloroplast transit peptide. The two SOUL protein genes induced by ^1^O_2_ in our study do not seem to be targeted to the chloroplast, and they may function in the cytosol where SAK1 resides. It would be interesting to test whether these proteins bind porphyrins and are required for ^1^O_2_ acclimation.

A recent study reported the role of bilins in retrograde signaling in *Chlamydomonas* through characterization of heme oxygenase mutants disrupted in bilin biosynthesis and transcriptome analyses during dark to light transitions (Duanmu et al., 2013). The transcriptome changes indicated that much of the cell’s response during a dark-to-light transition (DL) involves photo-oxidative stress. Interestingly, among the 515 genes up-regulated in WT during ^1^O_2_ acclimation, 144 genes overlapped with those that are induced during DL (Table 9). Focusing on the 104 genes that we defined as SAK1-dependent (Table 5), 31 genes overlapped (Table 9). *CFA1*, *CFA2*, and *SOUL2* were among these genes, suggesting that a part of the gene expression response to DL in *Chlamydomonas* is a response to ^1^O_2_. *SAK1* itself was also up-regulated during DL as was *SOR1*, which encodes a more broadly oxidative stress-responsive bZIP transcription factor (Fischer et al., 2012). We found that 64 of the genes induced during acclimation to ^1^O_2_ were also up-regulated in the gain-of-function *sor1* mutant (Fischer et al., 2012). However, the most strongly induced SAK1-dependent genes were not among these genes, except for *GPX5*, consistent with the idea that *SAK1* and *SOR1* function in different pathways.

**Table 9.** Genes up-regulated during both ^1^O_2_ acclimation and dark to light transition **DOI:** http://dx.doi.org/10.7554/eLife.02286.021

Gene ID (v4)	Gene name	Annotation	RB (log_2_)	DL (log_2_) (Duanmu et al., 2013)
Cre02.g137700.t1.1*			6.49	2.34
Cre06.g281250.t1.1*	*CFA1*	cyclopropane fatty acid synthase	5.92	4.49
Cre01.g033300.t1.1*			5.72	3.62
Cre13.g566850.t1.1*	*SOUL2*	SOUL heme-binding protein	5.53	2.25
Cre13.g600650.t1.1*			4.76	3.26
Cre06.g263550.t1.1*	*LCI7*	R53.5-related protein	4.46	5.27
Cre07.g342100.t1.1*			4.43	1.84
Cre09.g398700.t1.1*	*CPLD27*	coclaurine N-methyltransferase	4.05	1.36
Cre12.g492650.t1.1*	*FAS2*	fasciclin-like protein	4.01	9.24
Cre08.g381510.t1.1*			3.94	3.27
Cre10.g458450.t1.2*	*GPX5*	glutathione peroxidase	3.91	3.08
Cre11.g474600.t1.1*			3.90	1.99
Cre13.g600700.t1.1*			3.78	5.79
Cre14.g613950.t1.1*			3.65	2.68
Cre06.g269300.t1.1*			3.50	1.99
Cre08.g380300.t1.2*	*MSRA3*	peptide methionine sulfoxide reductase	3.45	1.79
Cre01.g031650.t1.2*	*CGLD12*	protein with potential galactosyl transferase activity	3.30	4.90
Cre14.g629061.t1.1*			3.25	1.88
Cre13.g564900.t1.1*			3.22	3.38
Cre13.g586450.t1.1			3.21	3.50
Cre02.g139500.t1.1*			3.04	2.12
Cre19.g756100.t1.1			3.04	6.53
Cre01.g036000.t1.2			3.02	1.16
Cre14.g618400.t1.1*			2.97	2.16
Cre17.g741300.t1.2*			2.88	1.92
Cre16.g648700.t1.2*			2.79	2.35
Cre17.g729950.t1.1			2.77	2.61
Cre17.g721000.t1.1			2.70	2.12
Cre06.g263500.t1.1*			2.67	3.37
Cre01.g016150.t1.1*			2.65	2.92
Cre08.g380000.t1.1*			2.59	3.74
Cre04.g224800.t1.1	*VAMP74*	R-SNARE protein, VAMP72-family	2.58	3.34
Cre03.g210150.t1.1			2.57	3.44
Cre14.g615600.t1.1*			2.53	2.40
Cre06.g293100.t1.1		Qc-SNARE SYP6-like protein	2.48	4.90
Cre08.g368950.t1.1	*DHQS*	3-dehydroquinate synthase	2.39	2.49
Cre10.g424350.t1.2		metalloprotease	2.37	3.18
Cre12.g537225.t1.1			2.34	3.39
Cre07.g336900.t1.2			2.32	2.31
Cre16.g664050.t1.1			2.31	1.88
Cre16.g677750.t1.1			2.04	2.22
Cre12.g537227.t1.1			2.00	3.46
Cre17.g737050.t1.1		RabGAP/TBC protein	1.99	2.32
Cre06.g297450.t1.1			1.93	1.46
Cre06.g258600.t1.1*			1.91	3.63
Cre16.g663950.t1.1		SC5D, C-5 sterol desaturase	1.89	2.03
Cre13.g588150.t1.1			1.86	6.21
Cre17.g722150.t1.1	*PKS3*	type III polyketide synthase	1.85	1.61
Cre16.g688550.t1.1	*GSTS1*	glutathione-S-transferase	1.84	1.20
Cre03.g207800.t1.1			1.84	7.09
Cre10.g444550.t1.1*	*SPP1A*	signal peptide peptidase	1.81	5.33
Cre13.g602500.t1.2			1.76	1.59
Cre03.g163400.t1.2*			1.76	2.15
Cre10.g450000.t1.1			1.74	2.18
Cre01.g015500.t1.1			1.72	1.55
Cre02.g105750.t1.2			1.71	3.23
Cre01.g061750.t1.1	*SPT2*	serine palmitoyltransferase	1.71	2.29
Cre83.g796250.t1.1			1.68	1.59
Cre16.g656150.t1.1			1.67	3.55
Cre01.g002050.t1.2			1.66	3.15
Cre12.g556750.t1.2	*Tic32-like 1*	Short-chain dehydrogenase, classical family, similar to PsTic32	1.66	3.15
Cre12.g559100.t1.1			1.66	3.11
Cre09.g411750.t1.2			1.61	1.96
Cre11.g482650.t1.2			1.57	3.40
Cre06.g310500.t1.1*			1.57	6.23
Cre09.g397900.t1.1		transmembrane protein	1.56	2.02
Cre04.g215600.t1.1			1.53	2.64
Cre02.g093800.t1.1			1.51	4.99
Cre02.g093750.t1.1	*NRX2*	Nucleoredoxin 2	1.50	6.26
Cre01.g004350.t1.1			1.50	2.29
Cre01.g034600.t1.1			1.50	2.22
Cre11.g472600.t1.2			1.48	2.00
Cre12.g500500.t1.2	*SMT1*	sterol-C24-methyltransferase	1.46	3.05
Cre13.g577950.t1.1	*VPS6*	subunit of the ESCRT-III complex	1.45	2.36
Cre02.g118200.t1.1			1.44	2.79
Cre01.g012500.t1.1	*PRA1*	prenylated rab acceptor family protein	1.43	2.46
Cre12.g521600.t1.2			1.42	2.89
Cre03.g179100.t1.1		ubiquitin fusion degradation protein	1.41	3.38
Cre09.g413150.t1.2			1.39	4.31
Cre13.g572200.t1.1		tyrosine/tryptophan transporter protein	1.39	2.57
Cre03.g185850.t1.2		PfkB-type carbohydrate kinase	1.37	3.05
Cre18.g743600.t1.1			1.37	1.65
Cre02.g076800.t1.1		sterol reductase	1.36	2.41
Cre06.g256750.t1.1	*FAT1*	acyl carrier protein thioesterase	1.35	1.67
Cre17.g729450.t1.1			1.34	1.90
Cre11.g471550.t1.1			1.34	3.29
Cre09.g395750.t1.2			1.33	2.87
Cre14.g617100.t1.1			1.33	3.33
Cre16.g691500.t1.1		Sec14p-like lipid-binding protein	1.33	2.28
Cre02.g079550.t1.1	*DRP2*	Dynamin-related GTPase	1.32	2.34
Cre02.g079300.t1.1	*VPS4*	AAA-ATPase of VPS4/SKD1 family	1.32	1.96
Cre05.g231700.t1.2			1.31	2.40
Cre02.g132300.t1.2	*DNJ12*	DnaJ-like protein	1.30	2.24
Cre69.g794101.t1.1			1.30	2.65
Cre13.g565600.t1.2			1.29	3.42
Cre13.g593700.t1.1		monooxygenase, DBH-like	1.29	1.81
Cre12.g498000.t1.2			1.28	3.88
Cre06.g292900.t1.2			1.28	2.16
Cre08.g372100.t1.1	*HSP70A*	Heat shock protein 7A	1.27	2.28
Cre01.g039350.t1.1	*NCR2*	NADPH-cytochrome P45 reductase	1.26	2.19
Cre03.g211100.t1.1			1.26	2.11
Cre17.g731800.t1.1			1.25	1.78
Cre17.g730650.t1.1			1.25	2.28
Cre02.g123000.t1.2			1.24	1.42
Cre05.g247700.t1.2			1.24	2.71
Cre08.g360800.t1.2		haloacid dehalogenase-like hydrolase	1.23	4.39
Cre07.g350750.t1.1	*PTOX1*	alternative oxidase	1.22	3.32
Cre17.g703750.t1.1			1.20	2.21
Cre06.g306041.t1.1			1.20	2.90
Cre02.g116650.t1.1			1.20	2.83
Cre08.g379400.t1.2			1.18	3.04
Cre16.g677000.t1.1	*HSP70E*	Heat shock protein 7E	1.18	2.50
Cre06.g283900.t1.1			1.18	5.24
Cre14.g626750.t1.1			1.17	4.12
Cre01.g010700.t1.1			1.16	2.10
Cre01.g002000.t1.2		predicted proteim	1.15	1.68
Cre04.g213150.t1.1			1.15	2.78
Cre16.g694250.t1.1			1.15	2.92
Cre05.g246400.t1.1			1.15	2.74
Cre02.g128450.t1.1			1.13	2.82
Cre03.g180250.t1.1		Myo-inositol-1-phosphate synthase	1.13	2.05
Cre03.g186150.t1.1			1.13	1.78
Cre02.g137800.t1.1			1.13	2.00
Cre11.g471500.t1.1	*MFT10*	predicted protein	1.11	1.40
Cre10.g435200.t1.1			1.10	2.13
Cre13.g593850.t1.2			1.10	3.91
Cre19.g754000.t1.2			1.10	2.33
Cre13.g593869.t1.1			1.10	3.90
Cre08.g377300.t1.2			1.09	3.27
Cre04.g225050.t1.2		predicted protein	1.09	3.55
Cre07.g330300.t1.1			1.08	2.22
Cre12.g500450.t1.2			1.08	3.00
Cre06.g262000.t1.1			1.08	1.87
Cre10.g441550.t1.2	*MAM3B*	predicted protein	1.07	1.54
Cre06.g249800.t1.1		unknown conserved protein	1.07	2.08
Cre01.g038250.t1.1	*SDC1*	serine decarboxylase	1.06	1.92
Cre44.g788200.t1.1			1.06	2.13
Cre08.g359200.t1.2			1.03	2.69
Cre05.g245950.t1.1	*DRP1*	Dynamin-related GTPase	1.03	2.15
Cre05.g234100.t1.1	*CYP745A1*	cytochrome P45	1.01	2.61
Cre07.g328700.t1.2			1.01	1.56
Cre10.g440250.t1.2			1.01	2.14
Cre17.g725200.t1.1		MDR-like ABC transporter	1.01	3.30
Cre82.g796100.t1.1			1.01	2.49

* Genes defined as SAK1-dependent in Table 4.

### SAK1 is a key intermediate component in the retrograde signaling pathway for ^1^O_2_ acclimation

Cloning of the *SAK1* gene revealed that it encodes a large previously uncharacterized phosphoprotein located primarily in the cytosol (Figure 6A,D), suggesting that it functions as an intermediate in the retrograde signaling pathway from the chloroplast to the nucleus that leads to ^1^O_2_ acclimation. Previous genetic screens in *Arabidopsis* have identified proteins in the chloroplast, such as EX1 and EX2 (Wagner et al., 2004; Lee et al., 2007), and in the nucleus, such as PLEIOTROPIC RESPONSE LOCUS 1 (Baruah et al., 2009b) and topoisomerase VI (Simková et al., 2012), that are involved in ^1^O_2_ signaling. By screening for mutants that are unable to induce a ^1^O_2_-responsive reporter gene (*HPS70A*) in *Chlamydomonas*, a small zinc finger protein (Cre09.g416500.t1.2) called MBS was recently identified as having a role in ROS signaling in both *Chlamydomonas* and *Arabidopsis* (Shao et al., 2013). Like SAK1, MBS in *Chlamydomonas* is located in the cytosol, raising a question about the relationship of these two proteins in ^1^O_2_ signaling. As expected, we found *HSP70A* among the genes induced by RB treatment of *Chlamydomonas* (Table 3) however in *sak1* it was not significantly induced above the twofold threshold, suggesting that SAK1 might function in the same signaling pathway as MBS. The *MBS* gene itself is not induced by ^1^O_2_ (Shao et al., 2013), and we will investigate the genetic and biochemical relationship of SAK1 and MBS in future research.

SAK1 contains a novel domain of ∼150 amino acid residues that is found in several chlorophyte species (Table 8). The sequence of this domain is not highly conserved (Figure 5—figure supplement 1), and is even less conserved among land plant proteins, although it is detectable by PSI-BLAST, indicating that it has diverged in sequence in plants and algae. We identified 37 proteins that have the SAK1 domain, 13 of which also contained a bZIP transcription factor domain, consistent with a function in regulating gene expression. Under our standard laboratory growth conditions, SAK1 appears to have a relatively low level of phosphorylation, but it becomes hyperphosphorylated during ^1^O_2_ acclimation (Figure 6D). Phosphorylation prediction software NetPhos 2.0 (http://www.cbs.dtu.dk/services/NetPhos/) predicted 24 serine, 9 threonine, and one tyrosine residue as possible sites throughout the protein (Figure 5—figure supplement 3). One of these serine residues is within the conserved SAK1 domain and is relatively conserved for polar amino acids. At this position, 18 SAK1 family members had threonine, and three had serine residues including SAK1 (Figure 5—figure supplement 1). We speculate that phosphorylation of SAK1 in the cytosol is a necessary intermediate step in ^1^O_2_ acclimation. Through further analysis of the transcriptome data, isolation of proteins that physically interact with SAK1, and characterization of additional, non-allelic *sak* mutants, we hope to identify the kinase that is responsible for the direct modification of SAK1 as well as other upstream and downstream components of this retrograde signaling pathway in *Chlamydomonas*.

## Material and methods

### *Chlamydomonas* strains and culture conditions

The *sak1* mutant was generated by insertional mutagenesis as described previously (Dent et al., 2005) from WT strain 4A+. Cells were grown at 22°C photoheterotrophically in Tris-acetate phosphate media (TAP) unless otherwise stated (Harris, 2009).

### RB sensitivity screen and acclimation assays

For systematic screening of large number of strains for increased or decreased resistance to RB, individual strains were inoculated into 180-200 μl TAP medium in 96-well plates, grown for a at least 3 days to saturation under light intensity of 60–80 μmol photons m^−2^ s^−1^, spotted onto TAP plates with 2.7, 3.0, or 3.3 μM RB, and scored for their growth compared to WT and *sak1*. For more quantitative evaluation of RB sensitivity, the cells were grown to saturation in 1 ml of TAP medium because we have observed rapidly growing cells to have more variable sensitivity to RB (data not shown). The cells were counted and adjusted to equal cell density then dispensed into aliquots in duplicate 96-well plates. One of the duplicates was pretreated in dark while the other was placed in light for 40 min with 1 μM RB. For challenge treatments, 4.5, 5.1, 5.7, 6.3, 6.9, and 7.5 μM RB was added to both plates, which were placed under light for 1 hr and then spotted onto TAP agar media with no RB. All treatments were applied under light intensity of 60–80 µmol photons m^−2^ s^−1^, which is the light intensity described as low light unless stated otherwise.

### Pretreatment and challenge with RB and F_v_/F_m_ measurement

Cells were grown under 100 μmol photons m^−2^ s^−1^, adjusted to 2 × 10^6^ cells ml^−1^, and treated with RB at a final concentration of 0.5 μM for 30 min (pretreatment) in light (+) or dark (−). After the pretreatment all the cultures were exposed to an additional 3.75 μM RB (challenge) in low light and collected for measurement of F_v_/F_m_ at 30, 60, and 90 min. The cells were dark-acclimated for at least 30 min before applying a saturating light pulse of 2000 μmol photons m^−2^ s^−1^ and measuring the chlorophyll fluorescence yield using an FMS2 fluorometer (Hansatech Instruments, Norfolk, UK).

### Culture conditions for gene expression analyses by qRT-PCR and RNA-seq

Cultures were grown for at least two light–dark cycles (12 hr light-12 hr dark), and then cell density was adjusted to 2–2.5 × 10^6^ cells ml^−1^ and split into two flasks (one control and the other for RB treatment) at least an hour prior to adding RB to a final concentration of 1 μM. An equal volume of H_2_O was added to the control. RB was added ∼6 hr after the start of the light cycle under light intensity of ∼100 µmol photons m^−2^ s^−1^ and the treatment lasted for an hour before harvest. The cells were cooled and harvested by centrifugation at 1200×*g* for 3 min at 4°C, frozen with liquid nitrogen and stored at −80°C until extraction of RNA. For low light to high light transfer experiment, cultures were grown in continuous light in minimal (HS) medium for 3 days to cell density of 3 × 10^6^ cells ml^−1^ at 45 µmol photons m^−2^ s^−1^. The light intensity was increased to 500 µmol photons m^−2^ s^−1^ for 1 hr before harvest.

### Gene expression analysis by qRT-PCR

RNA was extracted with TRIzol (Life Technologies, Carlsbad, CA) following manufacturer's instructions and treated with DNaseI (Promega, Madison, WI), then cleaned up using Qiagen RNeasy columns (Qiagen, Germantown, MD). cDNA was synthesized using Omniscript (Qiagen, Germantown, MD) starting with 2–3 μg DNA-free RNA per 20 μl reaction. qPCR was performed using a 7300 FAST qPCR machine (Life Technologies, Carlsbad, CA). The primers were designed with a T_m_ of 60°C using Primer3 or PrimerExpress (Life Technologies, Carlsbad, CA) (Table 10). All primer pairs described in this study were confirmed as having 90–105% amplification efficiency and linear amplification within their dynamic range in experimental samples using serial dilutions of cDNA prior to the experiments. Relative transcript levels were calculated by ΔΔCt method (Livak and Schmittgen, 2001) using *CβLP* as internal reference.

**Table 10.** Primers used for qRT-PCR analyses **DOI:** http://dx.doi.org/10.7554/eLife.02286.022

v4 ID	v5 ID	Gene name	Forward	Reverse
Cre01.g007300.t1.1	Cre01.g007300.t1.2		AGCATGTGCGTGTGGAGTAG	CCTTACCATAGGCCTGACCA
au5.g10700_t1a	Cre03.g177600.t1.3		CTGGACATGTCGGCTATGAA	GCTCATGTCGTACTCCAGCA
au5.g13389_t1*	Cre06.g299700.t1	*SOUL1*†	TGCGTATGGGTGTCCACTAA	TGGGGATCTTCTTCATGTCC
Cre06.g263550.t1.1	Cre06.g263550.t1.2	*LCI7*	TTTGGTTGCGTTGCATGTAT	TCAACGCGGTGTCAAACTTA
Cre06.g281250.t1.1	Cre06.g281250.t1.2	*CFA1*	CCTACAACGACAACGACGTG	GGAAGTTCCAGGATGACCAG
Cre06.g298750.t1.1	Cre06.g298750.t1.2	*AOT4*	CCGTGTGCACAGATTCAAAG	CACACAGCGCCTCCTACATA
Cre08.g358200.t1.2	Cre08.g358200.t2.1		TGTGGCATCAAGGTGTGTTGT	AACCCCACACCCCTCTCTTT
Cre09.g398700.t1.1	Cre09.g398700.t1.2	*CFA2*	CGACCTGCTGCTCTACTTCC	GTGTAGGCGGTGGTCAAGAT
Cre10.g458450.t1.2	Cre10.g458450.t1.3	*GPX5*	AACCAATCGCCTAACACCTG	CACTTGCTAGCCACGTTCAC
Cre12.g503950.t1.1	Cre12.g503950.t1.2		GGAGGGAGTACCACGAGACA	GATTGCTGTAAGGCCGGATA
Cre13.g564900.t1.1	Cre13.g564900.t1.2	*MRP3*	TCATGACGTACATCTCGATTCTCA	AGGGAATGTAGTAGCGCTGAATG
au5.g4402_t1*	Cre13.g566800.t1.2		TGCTTGGAAGACCCACTTTT	GAGCTGGAGTTGCAGTTGTG
Cre13.g566850.t1.1	Cre13.g566850.t1.2	*SOUL2*	CCCTCCCCTCCTTCAGACTA	CGTACCTGAGGCGCATATTT
Cre14.g613950.t1.1	Cre14.g613950.t2.1		CGCCCAACCCCATGATC	CCGCAACGTACCGTGATG
Cre16.g683400.t1.1	Cre16.g683400.t1.2		CCTGAACAAACACACGATGG	GAACGCCGTCAAATCATCTT
Cre16.g688550.t1.1	Cre16.g688550.t1.2	*GST1*	AGTGCGGAGGAAGTCGTAAA	GTAAAAGACGTGCGTGCAAA
	g6364.t1	*CβLP(RCK1)*	GAGTCCAACTACGGCTACGC	GGTGTTCAGGTCCCACAGAC
Cre14.g623650.t1.1	Cre14.g623650.t1		GACAACGCGGCCTACAAGA	CCGAGCTGGCGGTGTTAA
au5.g2281_t1*	g16723.t1	*MKS1*	GCTTGAGCGCGAGACGAA	CGCTGAAAGCATTGCAGAAG
Cre08.g380300.t1.2	Cre08.g380300.t1.2		ACCACCAGCAGTACCTGTCC	CGCTCCAATAAAGCCTTCAG
au5.g7871_t1‡	(Cre17.g741300.t1.2)‡	*SAK1(5'UTR)*	CAAGTGCTCATGAGAGGCCTTA	TACGTCATCCAGTTCCACATCC
au5.g7871_t1‡	(Cre17.g741300.t1.2)‡	*SAK1(3'UTR)*	TCAAGCGTGTGGGTAAGAGCTA	ACGCTATCTCCGTCCTAATCCA
Cre08.g365900.t1.1	Cre08.g365900.t1.2	*LHCSR1*	CACACAATTCTGCCAACAGC	ATCTGCTTCACGGTTTGGTC
Cre04.g220850.t1.1	Cre04.g220850.t1.2		TAATGGTATGGATGCGGTCA	ACTGCCAGTTATGGGTCCTG
Cre09.g395750.t1.2	Cre09.g395750.t1.3		ACCGTCCGTGAACCTTACTG	CGCAAACACGTCTCAAAGAA

* Was originally mapped and identified as augustus version 5 models within *Chlamydomonas* genome v4.

† *SOUL1* was given the name in v4 but not v5.

‡ Primers were designed against experimentally obtained cDNA (Genbank accession KF985242) and differs from v5. Closest gene model is au5.g7871_t1.

### RNA-seq library preparation and analysis

RNA was extracted (Schmollinger et al., 2014) and the quality was determined using a 2100 Bioanalyzer (Agilent Technologies, Santa Clara, CA). The triplicate RNA was pooled and 10 μg total RNA was used to prepare RNA-seq library according to the manufacturer's protocol (Illumina, San Diego, CA). The quality of the library was assessed using a 2100 Bioanalyzer before sequencing with Genome Analyzer (Illumina, San Diego, CA). Each sample was run in replicates on two lanes. RNA-Seq data was analyzed as before (Duanmu et al., 2013). On average, 75% of the sequences could be assigned unambiguously to Augustus v10.2 gene models to generate the matrix of counts per gene. This matrix was used for differential expression analysis using *DESeq* (Anders and Huber, 2010) using *per-condition* dispersion estimates and variance stabilization to compute moderate fold changes. Genes were classified as differentially expressed based on a (moderate) twofold regulation and a false discovery rate (FDR) <1%.

### Amplification of cDNA and genomic region of *SAK1* and transformation of *sak1*

Near full-length cDNA was isolated by RT-PCR (described in above section; Gene expression analysis by qRT-PCR) and rapid amplification of cDNA ends (RACE) using GeneRACER (Life Technologies, Carlsbad, CA) as previously described (Molnar et al., 2009). Despite multiple attempts the 5′ end of the transcript could not be amplified by 5′-RACE. Because the experimentally obtained CDS differed from the most current v5, it has been deposited to genbank (accession KF985242). Though some differences exist at the nucleotide level, the protein sequence of the resulting CDS was identical to that of au5.g7871_t1. Genomic DNA containing *SAK1* was amplified using primers 5′-CAGGACCGGGCACTGAGTGAAGGTTA-3′ (+) and 5′-ATGATGCACTGTGGGACACGCTGAGT-3′ (−) using PrimeStar HS with GC buffer (Takara/Clontech, Palo Alto, CA) and cloned into pGEM-Teasy after adding an adenine. The resulting plasmid was co-transformed with pBC1 and selected with 1 μM paromomycin. Transformation of *sak1* was performed as described previously (Kindle et al., 1989).

### SAK1 antibody generation and protein detection by immunoblotting

To raise antibodies against SAK1, an epitope at the N-terminus of the translated coding sequence of SAK1 (DTLLTPLREDATAESGGDA) was designed, synthesized and injected into rabbits, and the resulting crude serum was affinity purified (Open Biosystems/Thermo Scientific, Waltham, MA). For immunoblot detection of SAK1, proteins were separated with NuPAGE 3–8% Tris Acetate gels (Life Technologies, Carlsbad, CA) and transferred to nitrocellulose membranes. All other blots were prepared from running the protein on 10–20% Tris-glycine gels and transferring to a PVDF membrane. The membranes were blocked for several hours in 5% milk in TBS-T, incubated with the primary antibody overnight, then with secondary antibody for several hours in 1% milk TBS-T before washing and developing with a chemiluminescence detection kit. Commercial antibodies were anti-histone H3 (ab1791; Abcam, Cambridge, UK) and anti-KDEL (ab12223; Abcam, Cambridge, UK). Other antibodies were generous gifts from Jean-David Rochaix (anti-PSAD), Olaf Kruse (anti-NAB1), and Patrice Hamel (anti-cytochrome *c*).

### Subcellular fractionation and protein quantification

Nuclear fractions were prepared from 450 ml of synchronized cultures with ∼2 × 10^6^ cells ml^−1^ that had been incubated with or without 2 μM RB under light for 40 min. The cells were collected and treated with autolysin for 40 min and examined for the removal of cell walls by addition of 1 volume of 0.1% Triton-X. Nuclear extract was prepared as described previously (Winck et al., 2011) using CelLytic PN kit (Sigma-Aldrich, St. Louis, MO). Because there were bands detected in the nuclear extract close to the size of SAK1, nuclear extract was prepared from WT (4A+) and *sak1* rather than a cell wall-deficient strain (*cw15*). Chloroplasts were isolated from cell wall-less strain *cw15* as described previously (Klein et al., 1983). Mitochondria were isolated as described (Eriksson et al., 1995). After unbroken cells, chloroplasts, and mitochondria were collected, the ER fraction was collected by centrifugation at 100,000×*g* for 90 min at 4°C. The remaining supernatant was enriched for cytosol. Protein was extracted and prepared for SDS-PAGE as described (Calderon et al., 2013) with minor modifications. Protein was quantified by using BCA1 kit (Sigma-Aldrich, St. Louis, MO) after extraction with the methanol-chloroform method (Wessel and Flügge, 1984).

## Additional files

Supplementary file 1. Genes that display significant differential expression by pair-wise comparisons. **DOI:** http://dx.doi.org/10.7554/eLife.02286.023

### Major dataset

The following dataset was generated:

WakaoS, ChinBL, LedfordHK, DentRM, CaseroD, PellegriniM, MerchantSS, NiyogiKK, 2014, Data from: Phosphoprotein SAK1 is a regulator of acclimation to singlet oxygen in Chlamydomonas reinhardtii, http://dx.doi.org/doi:10.5061/dryad.h7pm2, Available at Dryad Digital Repository under a CC0 Public Domain Dedication.
